# Supplementary material for: Efficacy of oligodendrocyte precursor cells as delivery vehicles for single-chain variable fragment to misfolded SOD1 in ALS rat model
Source: Mol Ther Methods Clin Dev. 2023 Feb 4;28:312–29. doi: 10.1016/j.omtm.2023.01.008 (PMC9974989; doi:10.1016/j.omtm.2023.01.008)
Supplement: Document S1. Figures S1–S5 and Tables S1 and S3–S6 [file mmc1.pdf]

OMTM, Volume *28*

Supplemental information

Efficacy of oligodendrocyte precursor cells as delivery vehicles for single-chain variable fragment to misfolded SOD1 in ALS rat model

Sumio Minamiyama, Madoka Sakai, Yuko Yamaguchi, Makiko Kusui, Hideki Wada, Ryota Hikiami, Yoshitaka Tamaki, Megumi Asada-Utsugi, Akemi Shodai, Akiko Makino, Noriko Fujiwara, Takashi Ayaki, Takakuni Maki, Hitoshi Warita, Masashi Aoki, Keizo Tomonaga, Ryosuke Takahashi, and Makoto Urushitani

A

B

C

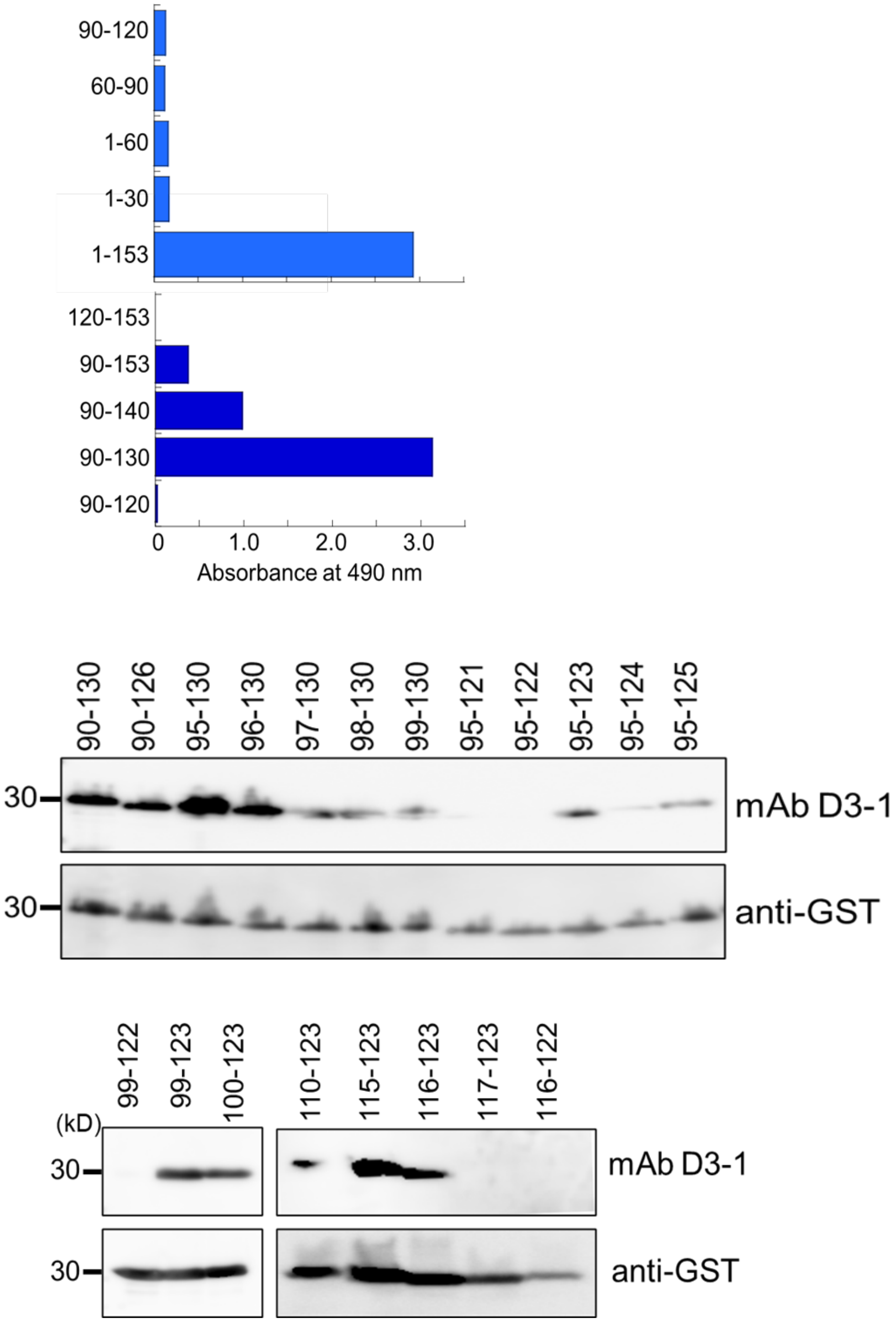

Figure S1. Epitopes of D3-1 exist within residues 116-123 of SOD1. **(A)** ELISA for GST-tagged deleted SOD1 peptides. **(B)** **(C)** Immunoblotting of *E. coli* extracts expressing GST-tagged deleted SOD1 peptides.

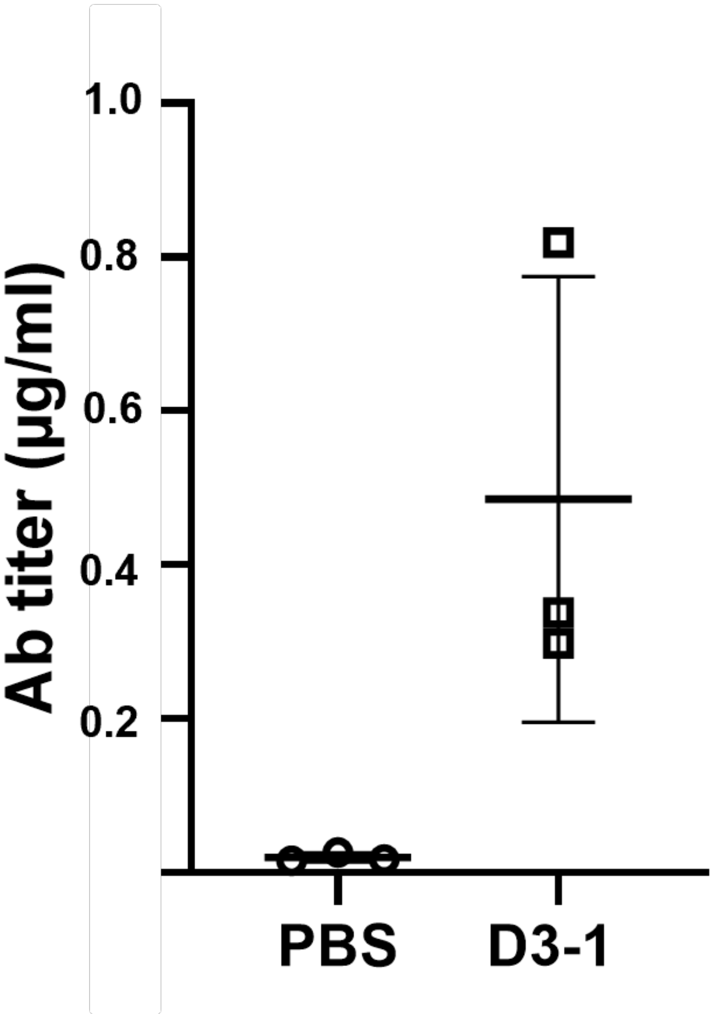

Figure S2. The titer of D3-1Mab in the D3-1 treated wild-type rats' CSF after 4 weeks was confirmed by an enzyme-linked immunosorbent assay (ELISA). ELISA for a full-length D3-1 in CSF from treated and PBS-injected controls was conducted to calculate the titer of antibodies. Differences were evaluated by student t-test (mean ± SD from three independent experiments; * $P < 0.05$).

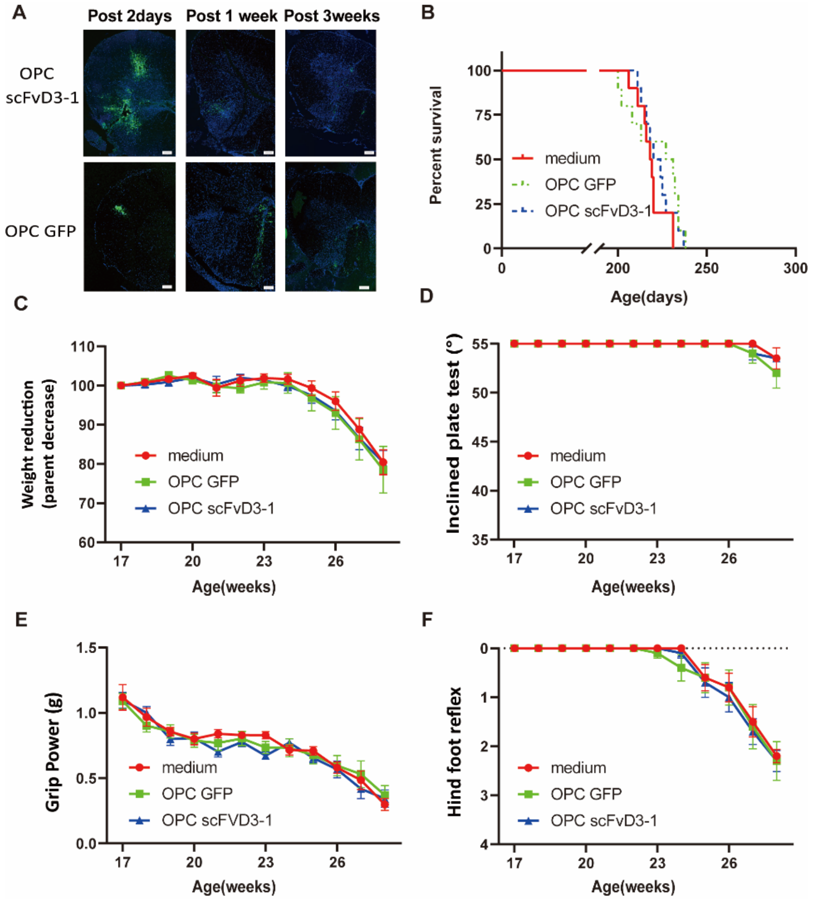

Figure S3. Single parenchymal injection of OPC scFvD3-1 had no therapeutic effect on disease onset and lifespan of SOD1H46R rats. (A) Immunohistochemical images of the lumbar spinal cord of OPC scFvD3-1 or OPC GFP injected rat two days, one week, and three weeks after injection showed a lot of injected alive OPCs two days after injection; however, only a few remained alive one and three weeks after injection. Scale bar 250 µm. (B) Parenchymal administration of OPC scFvD3-1 did not increase the lifespan of SOD1H46R rats. Kaplan–Meier curve for survival is shown (n = 10 for all $P > 0.05$ by log-rank test). (C) No reduction in body weight loss ($P > 0.05$); OPC scFvD3-1 treated and OPD GFP–treated rats, and medium-treated mice are shown in blue, green, and red respectively. (D) Inclined plate test, (E) forelimb grip power and (F) hindfoot reflex score: the score on each test was determined for SOD1H46R rats injected with OPC scFvD3-1, OPC GFP, and with medium (N =10 for all). OPC scFvD3-1 treatment did not improve motor performance ($P > 0.05$ by post hoc test) when compared with OPC GFP or medium–administered rats. Each point indicates average ± SEM. Differences were evaluated by two-way ANOVA.

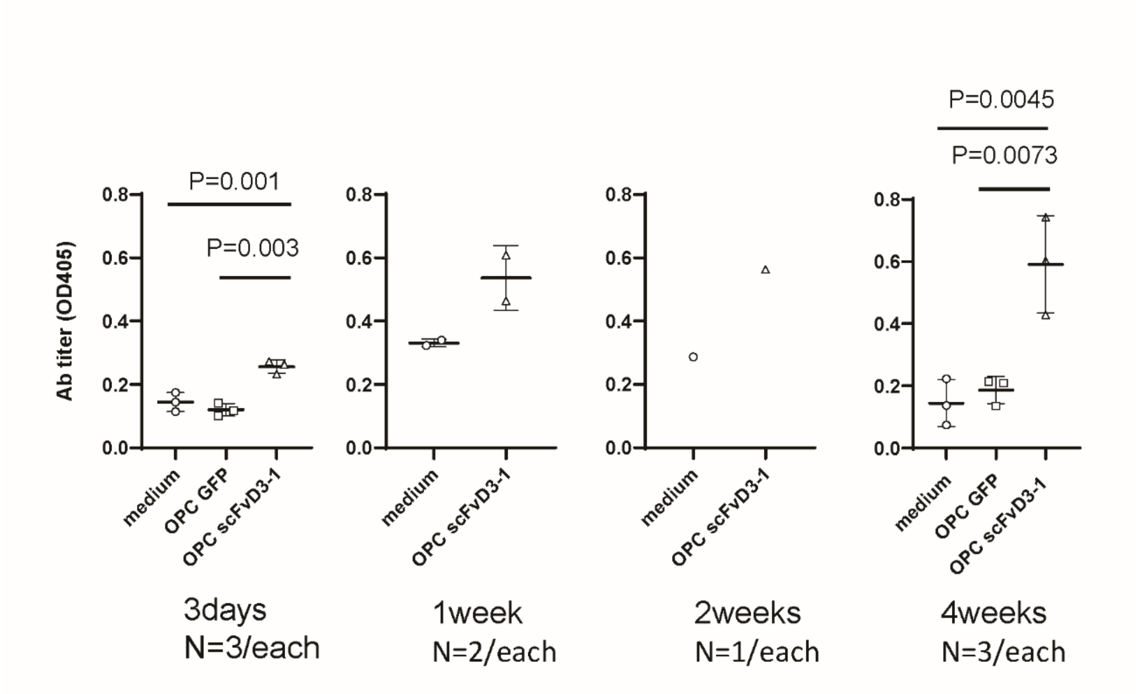

Figure S4. The relative amount of scFv in the cerebrospinal fluid of rats treated with OPC scFv D3-1 increases over time. The concentration of scFvD3-1 in the cerebrospinal fluid at three days one week, two weeks and four weeks after administration of OPC scFvD3-1, OPC GFP or medium, was measured by sandwich ELISA and found to be significantly higher in OPC scFvD3-1 treated rats than in OPC GFP or medium treated ones at three days and four weeks. After 1 and 2 weeks, the values were higher in the OPC scFvD3-1 treated rats than in the medium treated rats.

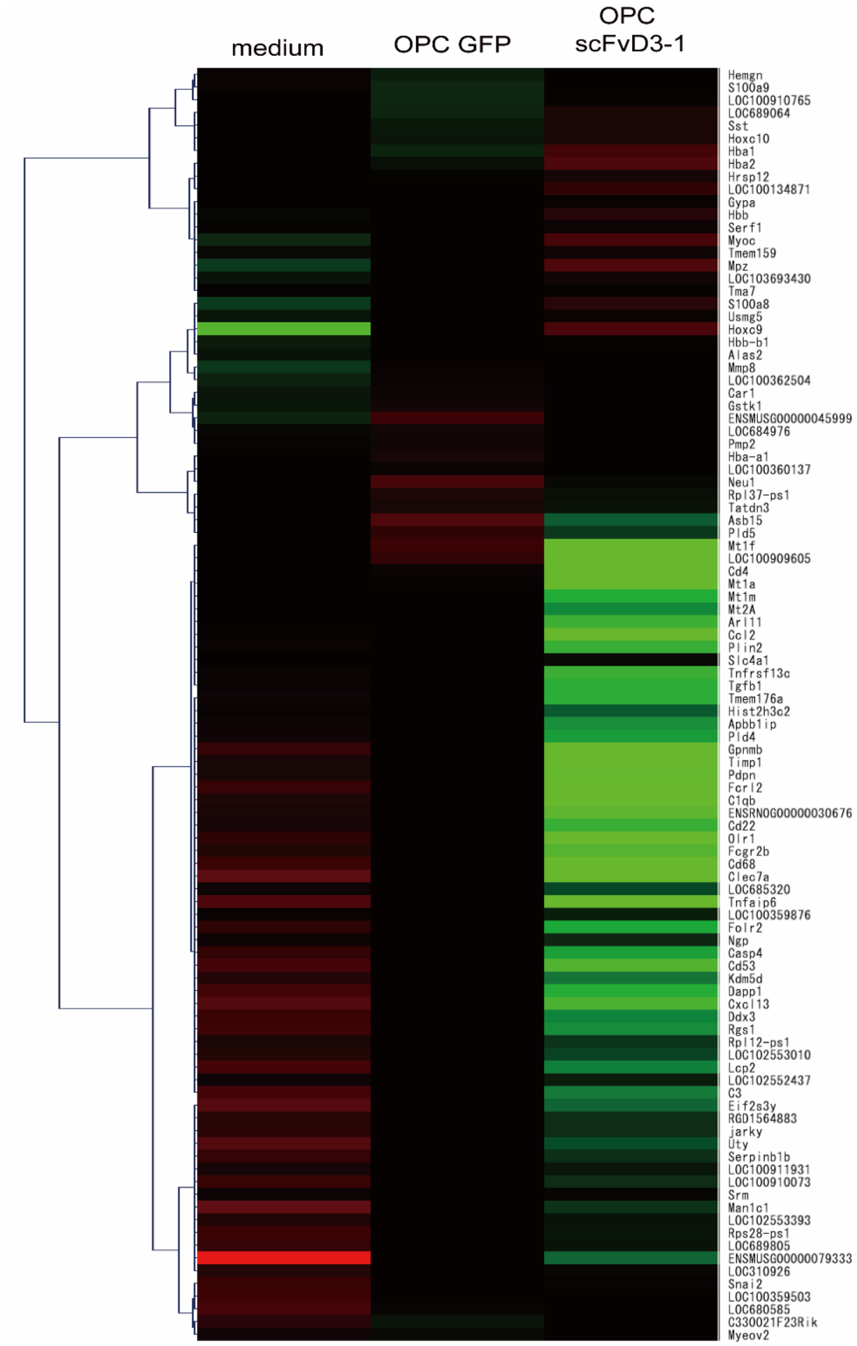

Figure S5. Heat map of microarray analysis using lumber spinal cord of SOD1H46R rats eight weeks after administration of medium, OPC GFP or OPC scFvD3-1(N=3). Green and red lines show decreased and increased genes respectively. The values indicate how far away from the median each of the three groups. The 100 genes with the highest value are listed. Wild-type rats were used as controls.

Table S1 Clinical information of ALS patients and control subjects.

Case no.	sex	Diagnosis	SOD1mutation	Age at death(years)	Duration of the disease
1	Male	mSOD1-ALS	I113T	64	7months
2	Male	mSOD1-ALS	I112T	43	2 years
3	Male	sALS		65	2 years
4	Male	sALS		79	1 years
5	Male	sALS		57	8 years 10 months
6	Male	sALS		65	1 year 11 months
7	Female	sALS		71	1 year 8months
8	Female	C(SCA)		63	
9	Female	C(DRPLA)		59	
10	Male	C(PD)		76	
11	Male	C(CI)		87	
12	Female	C(epilepsy)		51	

nd; not described, m; mutant, s; sporadic, C; control, SCA; spinocerebellar ataxia, DRPLA; dentatorubropallidoluysian atrophy, PD; Parkinson's disease, CI; cerebral infarction

Table S2 Motor function scores of rats after 29 weeks ages

Table S3 Gene profiles of altered expression levels

Decreased

Gene symbol	Signal value			Z score	
	medium	OPC GFP	OPC scFvD3-1	medium vs. OPCscFvD3-1	OPC GFP vs. OPCscFvD3-1
Gpnmb	6398.686	5409.877	235.688	-22.049	-26.058
Clec7a	2097.115	1232.396	72.245	-24.793	-24.343
Fcrl2	760.392	545.002	40.704	-21.537	-22.255
Cd68	1012.608	740.457	106.018	-16.581	-16.644
Tnfaip6	607.425	440.746	77.010	-15.167	-14.927
Ccl2	568.497	438.094	83.077	-14.117	-14.221
Olr1	513.551	421.769	89.707	-12.799	-13.232
Mt1a	12055.217	12279.805	2283.657	-10.641	-13.138
Mt1f	320.601	390.308	90.946	-9.217	-12.445
Tmem52b	180.636	112.667	51.997	-17.607	-12.413
C1qb	3065.082	2273.895	510.079	-11.951	-12.369
Cd4	20494.086	20494.086	4381.746	-9.864	-12.038
Ccl3	46.152	46.152	22.406	-10.187	-11.595
Mpeg1	4460.254	3880.186	997.905	-9.970	-11.227
Cd22	143.984	134.326	66.928	-10.804	-11.176
Csf2rb	113.498	113.498	56.974	-9.713	-11.055
Pdpn	17133.829	15036.279	3650.909	-9.886	-11.035
Cyp4v3	456.240	475.367	131.806	-9.082	-10.945
Glipr1	171.043	149.902	77.420	-5.765	-10.596
Grn	2582.495	2334.439	662.776	-9.051	-10.401
Cxcl13	2087.065	1229.444	369.978	-11.528	-9.914
Cd53	16982.250	11499.552	3278.295	-10.520	-9.770
Fcgr2b	2680.804	2082.918	643.658	-9.497	-9.693

Increased

Gene symbol	Signal value			Z score	
	medium	OPC GFP	OPC scFvD3-1	medium vs. OPCscFvD3-1	OPC GFP vs. OPCscFvD3-1
Igkc	93.805	78.997	391.847	10.653	13.922
Chodl	2249.268	3873.065	11973.356	10.765	9.026
Myh4	36.889	36.889	60.771	7.156	8.134
Slc18a3	742.445	1392.421	2945.323	9.283	6.376
Ahnak2	13863.462	14418.565	25476.156	3.809	5.349
Susd2	1381.567	1832.643	3399.696	6.086	4.999

Table S4 Antibodies used in the study

	Applications	Host	Source	catalog #
C4F6	WB	Mouse	Medimabs	#MM-0070-2
FLAG	WB,IF,IP	Mouse	Sigma-Aldrich	F1804
β-actin	WB	Mouse	Santa Cruz	sc-47778
Myc	WB,IF,IP,ELISA	Rabbit	Cell Signaling	#2278
GAPDH	WB	Rabbit	Santa Cruz	sc-25778
GFP	IF,IHC	Rabbit	MBL	598
chat	IHC	Goat	Millipore	AB144P
iba1	IHC	Rabbit	Wako	MNH4488
GFAP	IHC	Mouse	Sigma-Aldrich	032M4779
synapshin1	IHC	Mouse	Sigma-Aldrich	S5788
NG2	IF	Mouse	abcam	ab5009
α-Bungarotoxin	IHC		ThermoFisher	B13423
MCT-1	WB	Rabbit	Millipore	AB3540P

WB: western blot; IF: Immunofluorescence; IHC: immunohistochemistry;
IP: immunoprecipitation; ELISA: Enzyme-linked immunosorbent assay

Table S5 Primer Sequences for the construction

SOD1 I112M fwd	5’-TCA GGA GAC CAT TGC ATG ATT GGC CGC ACA C-3’
SOD1 I112M rev	5’-GTG TGC GGC CAA TCA TGC AAT GGT CTC CTG A-3’
SOD1 H46R fwd	5’-GGG GCT AGC CAC CAT GGC GAC GAA GGC CGT GTG-3’
SOD1 H46R rev	5’-CCC GGA TCC CGT TGG GCG ATC CCA ATT ACA C-3’
VH fwd	5’-GAC TCG AGT CGA CAT CGA TTT TTT TTT TTT TTT TT-3
VH rev	5’-CTC AAT TTT CTT GTC CAC CTT GGT GC-3’
VH fwd	5’-GAC TCG AGT CGA CAT CGA TTT TTT TTT TTT TTT TT-3’
VL rev	5’-CTC ATT CCT GTT GAA GCT CTT GAC AAT GGG-3’

Table S6 Primer sequences for quantitative real-time PCR

Gene name	Primer Sequence
Ccl2_F	CTATGCAGGTCTCTGTCACGCTTC
Ccl2_R	CAGCCGACTCATTGGGATCA
Pdpn_F	TGAGGCTCCAACGAGATCAAGA
Pdpn_R	CGCTGAGTCCCAGAACCAAAC
Cxcl13_F	AACTCCACCTCCAGGCAGAATG
Cxcl13_R	GGTCGAGCTCACCTTGGAACA
Fcgr2b_F	CAGCAACAGGACTGTCGTCCA
Fcgr2b_R	GCTCAAGTTTCACCACAGCCTTC
Olr1_F	GATGACAAGATGAAGCCTGTGAATG
Olr1_R	GGATGGCCAGAGTCACAGCA
Tnfaip6_F	CGTCTTGCAACCTACAAGCAGCTA
Tnfaip6_R	ACAGTTGGGCCCAGGTTTCA
GAPDH_F	CAAGTTCAACGGCACAGTCAAG
GAPDH_R	ACATACTCAGCACCAGCATCAC
