# Supplementary material for: Efficacy of oligodendrocyte precursor cells as delivery vehicles for single-chain variable fragment to misfolded SOD1 in ALS rat model
Source: Mol Ther Methods Clin Dev. 2023 Feb 4;28:312–29. doi: 10.1016/j.omtm.2023.01.008 (PMC9974989; doi:10.1016/j.omtm.2023.01.008)
Supplement: Document S2. Article plus supplemental information [file mmc3.pdf]

Molecular Therapy
Methods & Clinical Development
Original Article

Efficacy of oligodendrocyte precursor cells as delivery vehicles for single-chain variable fragment to misfolded SOD1 in ALS rat model

Sumio Minamiyama,[1,2] Madoka Sakai,[3,4] Yuko Yamaguchi,[2] Makiko Kusui,[1] Hideki Wada,[1] Ryota Hikiami,[1,2] Yoshitaka Tamaki,[1] Megumi Asada-Utsugi,[1] Akemi Shodai,[1] Akiko Makino,[3,4] Noriko Fujiwara,[5] Takashi Ayaki,[2] Takakuni Maki,[2] Hitoshi Warita,[6] Masashi Aoki,[6] Keizo Tomonaga,[3,4,7] Ryosuke Takahashi,[2] and Makoto Urushitani[1]

[1]Department of Neurology, Shiga University of Medical Science, Otsu, Shiga 5202192, Japan; [2]Department of Neurology, Kyoto University Graduate School of Medicine, Kyoto 6068507, Japan; [3]Laboratory of RNA Viruses, Department of Virus Research, Institute for Frontier Life and Medical Sciences (InFRONT), Kyoto University, Kyoto 6068507, Japan; [4]Department of Mammalian Regulatory Network, Graduate School of Biostudies, Kyoto University, Kyoto 6068507, Japan; [5]Department of Biochemistry, School of Medicine, Hyogo Medical University, Nishinomiya, Hyogo 6638501, Japan; [6]Department of Neurology, Tohoku University Graduate School of Medicine, Sendai, Miyagi 9808574, Japan; [7]Department of Molecular Virology, Graduate School of Medicine, Kyoto University, Kyoto 6068507, Japan

***Superoxide dismutase1* (*SOD 1*) mutation is a leading cause of familial amyotrophic lateral sclerosis (ALS). Growing evidence suggests that antibody therapy against misfolded SOD1 protein can be therapeutic. However, the therapeutic effects are limited, partly because of the delivery system. Therefore, we investigated the efficacy of oligodendrocyte precursor cells (OPCs) as a drug delivery vehicle of single-chain variable fragments (scFv). Using a Borna disease virus vector that is pharmacologically removable and episomally replicable in the recipient cells, we successfully transformed wild-type OPCs to secrete scFv of a novel monoclonal antibody (D3-1), specific for misfolded SOD1. Single intrathecal injection of OPCs scFvD3-1, but not OPCs alone, significantly delayed disease onset and prolonged the lifespan of ALS rat models expressing *SOD1* ^{H46R}. The effect of OPC scFvD3-1 surpassed that of a 1 month intrathecal infusion of full-length D3-1 antibody alone. scFv-secreting OPCs suppressed neuronal loss and gliosis, reduced levels of misfolded SOD1 in the spinal cord, and suppressed the transcription of inflammatory genes, including *Olr1*, an oxidized low-density lipoprotein receptor 1. The use of OPCs as a delivery vehicle for therapeutic antibodies is a new option for ALS in which misfolded protein and oligodendrocyte dysfunction are implicated in the pathogenesis.**

INTRODUCTION

Amyotrophic lateral sclerosis (ALS) is a lethal neurodegenerative disease characterized by progressive muscle weakness and respiratory failure. Approximately 10% of ALS cases are familial, and 90% are sporadic.[1] Mutation in the genes encoding superoxide dismutase 1 (*SOD1*) account for 20% of familial ALS cases.[2,3] Multiple treatment approaches have been tested in animal models of ALS, including pharmacological approaches, cell transplantation, and oligonucleotide therapy.[4–6] Similarly, various cell transplantation therapies have been tested *in vivo*.[6,7] Oligodendrocytes maintain the neuronal architecture and serve as an energy supplier to neurons through lactate monocarboxylate transporter (MCT). Recent evidence suggests the dysfunction and turnover acceleration of oligodendrocytes in ALS transgenic (Tg) mice. Hence, oligodendrocyte precursor cells (OPCs) are likely to be involved in the pathogenesis of ALS from an early stage.[8,9] Therefore, OPCs are potential candidates for cell transplantation in ALS.

Protein misfolding underlies the molecular basis of ALS. The elimination of these pathogenic proteins, specifically by antibodies, is an ideal and rational therapeutic strategy. The efficacy of vaccination or antibody infusion has been reported against misfolded SOD1 proteins in rodent models.[5,10–12] However, the efficacy was limited, and further modification can improve the effect. In this regard, antibody delivery is a crucial factor that can augment the effect because the infusion of antibodies results in diffusion in cerebrospinal fluid (CSF) or entrapment in systemic tissues. Therefore, we tested a combination therapy of OPC transplantation and an antibody targeting extracellular SOD1, using a single chain of variable fragments (scFv) derived from a monoclonal antibody against misfolded SOD1.

Vector choice is crucial when using transformed cells in the clinical setting. As OPCs are dividing cells, gene delivery vectors should be replicable and should assemble with the host DNA. Additionally, host genes should not be affected by the vectors, such as chromosome integration in lentivirus. Adeno-associated

Received 26 February 2022; accepted 31 January 2023;
https://doi.org/10.1016/j.omtm.2023.01.008.

Correspondence: Makoto Urushitani, MD, PhD, Department of Neurology, Shiga University of Medical Science, Tsukinowa, Otsu, Shiga 5202192, Japan.
E-mail: uru@belle.shiga-med.ac.jp

 © 2023 The Author(s).
This is an open access article under the CC BY license (http://creativecommons.org/licenses/by/4.0/).

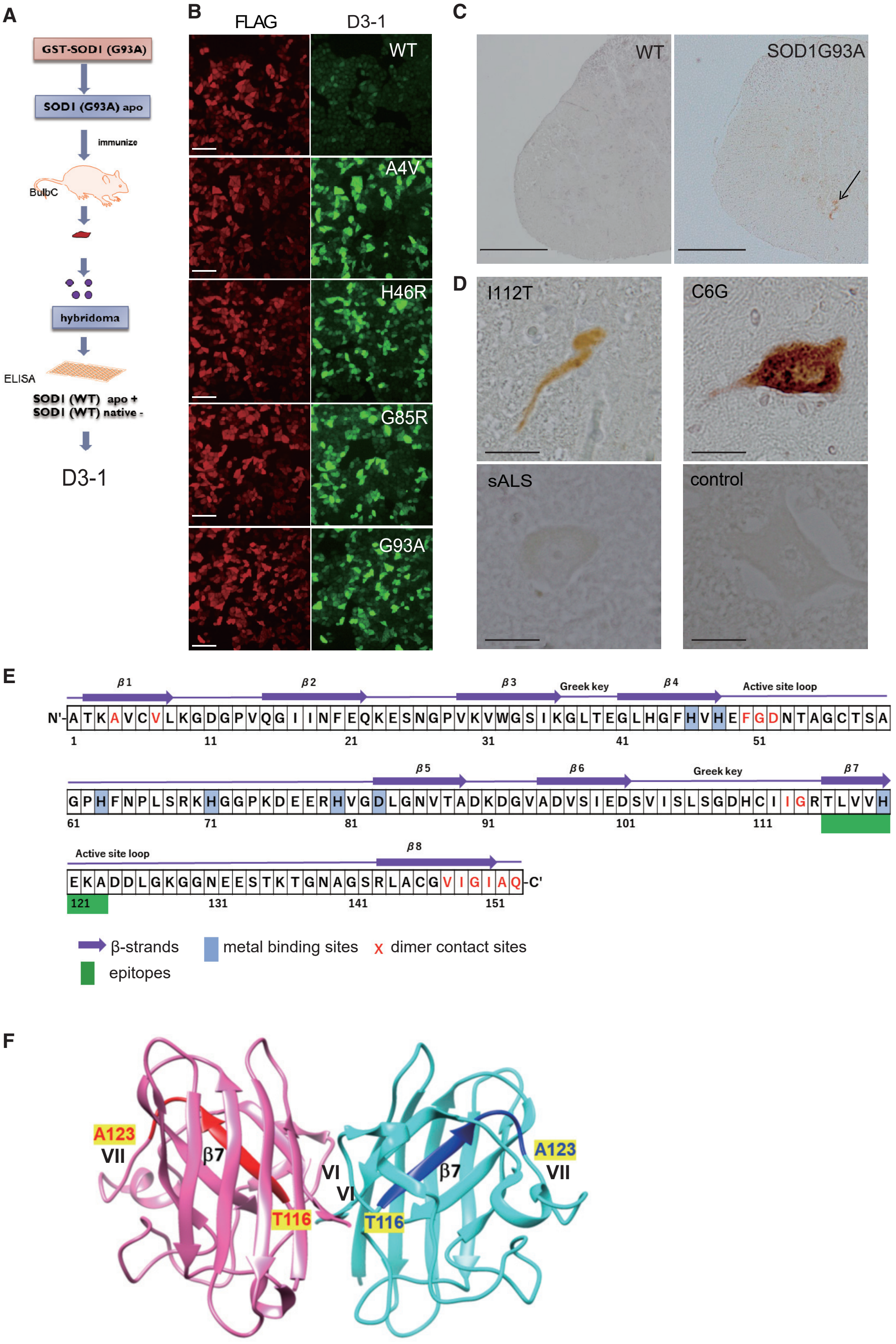

(legend on next page)

virus (AAV) vectors are a safe and effective modality already used clinically, but they do not replicate. Borna disease virus (BoDV) is a non-segmented negative-strand RNA virus that establishes persistent infection in the nucleus.[13] BoDV stays episomally inside the nucleus, with no concern for chromosomal integration. Viral ribonucleoproteins bind and segregate with host chromosomes during cell division.[14] Another advantage of BoDV is its removability; the antiviral drug favipiravir can eliminate the virus.[15,16] We have developed an RNA virus-based episomal vector (REVec) as a non-integrating stable gene expression system based on the features of BoDV.[16]

In this study, we report the high therapeutic potential of novel immunotherapy using OPCs expressing therapeutic scFv against toxic SOD1 proteins in ALS rat models.

RESULTS

Generation of the D3-1 antibody broadly targeting misfolded SOD1

The D3-1 monoclonal antibody (D3-1 mAb) was produced using a standard immunization protocol after immunizing mice with the recombinant demetalated (apo) SOD1^{G93A} protein,[17] followed by hybridoma screening through positivity to apo SOD1^{WT} and negativity with native SOD1^{WT} (Figure 1A). Positive selection of apo-wild-type (WT) SOD1 was based on previous notions, including ours, that apo-WT structurally mimics familiar ALS (FALS) SOD1 mutants.[18] The second screening used cell culture studies transfected with FLAG-fused WT and mutant SOD1 plasmids. A double immunofluorescence study using HeLa cells tested mAb clones for binding to several kinds of SOD1 mutants but not with WT. On the basis of multiple confocal microscopic evaluations, we selected one clone, D3-1, that recognized SOD1^{A4V}, SOD1^{H46R}, SOD1^{G85R}, SOD1^{G93A}, and SOD1^{I112M}, but not SOD1^{WT} (Figure 1B). Immunohistochemistry (IHC) of spinal cords from *SOD1^{G93A}* and *SOD1^{WT}* mice littermates also displayed similar results, in which D3-1 recognized only SOD1^{G93A} but not WT (Figure 1C). D3-1 reacted with several mutant types of SOD1 proteins in spinal cord sections from patients with FALS. In contrast, D3-1 did not recognize SOD1 in patients with sporadic ALS or other neurodegenerative diseases (Figure 1D; Table S1). Epitope mapping using various recombinant SOD1 fragments (Figure S1) revealed that the epitope of D3-1 exists within residues 116–123, covering β7 and a part of active site loop (Figures 1E and 1F).[19] In particular, Thr116 and Ala123 are crucial epitopes for D3-1 (Figure S1C).

Four week intrathecal infusion of D3-1 full-length mAb improved the phenotypes and extended the lifespan of *SOD1^{H46R}* rats

Before stepping forward to our ultimate goal of using scFv, we investigated the therapeutic potential of D3-1 mAb against extracellular SOD1 proteins. As a disease model, transgenic rats with *SOD1^{H46R}* mutation were chosen. The *SOD1 ^{H46R}* rats we generated have a slower onset and progression rate than *SOD1^{G93A}* transgenic rats. It should be noted that interindividual variation is small among the same genotype, allowing us to assess the therapeutic effect consistently.[20,21] Patients with ALS and *SOD1^{H46R}* are slowly progressive; however, the phenotype of transgenic *SOD1^{H46R}* rats is an appropriate model for our strategy. The larger population of patients with ALS and *SOD1^{H46R}* also promoted us to use this genotype. Using this model, we tested the therapeutic effect of full-length D3-1 mAb (FL D3-1) intrathecally administered by an osmotic pump for 4 weeks at 19 weeks, approximately 2–4 weeks before onset (Figure 2A). D3-1 mAb significantly extended lifespan by 15 days compared with phosphate-buffered saline (PBS) infusion (213.5 ± 6.9 days in PBS-treated rats versus 228.5 ± 16.7 days in D3-1-treated rats, n = 12, p = 0.0127) (Figure 2B). The onset, determined as the beginning of body weight loss, was significantly delayed by 1.5 weeks. Body weight measurements revealed a significantly slower loss in D3-1-treated transgenic rats from 27 to 28 weeks than PBS-treated transgenic rats ($p < 0.001$; mean 74.13% ± 17.31% in D3-1-treated rats versus 59.21% ± 9.90% in PBS-treated rats at the age of 28 weeks) (Figure 2C). The inclined plate test revealed that D3-1-treated animals could stay significantly longer, indicating preserved hindlimb strength compared with those treated with PBS ($p < 0.0001$; mean 52.08° ± 5.94° in D3-1-treated rats versus 41.25° ± 8.20° in PBS-treated rats at the age of 28 weeks) (Figure 2D). The grip strength test showed that D3-1-treated rats had a significantly slower decline in forelimb muscle strength than PBS-injected ones ($p < 0.05$; mean 0.3947 ± 0.280 g in D3-1-treated rats versus 0.1327 ± 0.066 g in PBS-treated rats at the age of 28 weeks) (Figure 2E). Reflex scores, the functional scale of upper motor neurons, demonstrated that D3-1-treated rats showed significantly higher reflex scores from 26 to 28 weeks of age compared with those treated with PBS ($p < 0.001$; mean 2.167 ± 1.280 in D3-1-treated rats versus 3.273 ± 0.850 in PBS-treated rats at the age of 28 weeks) (Figure 2F). The motor function scores of D3-1-treated rats showed slower progression than those of PBS-treated rats after 29 weeks of age (Table S2). The presence of D3-1 mAb in the CSF after four weeks was confirmed by using an enzyme-linked immunosorbent assay (ELISA). The results showed that the D3-1 mAb remained in the CSF from the Tg

Figure 1. Generation of monoclonal antibody (D3-1) against misfolded superoxide dismutase 1 (SOD1)

(A) Schematic diagram of D3-1 generation. The D3-1 monoclonal antibody was produced using standard procedures after immunizing mice with rec apo G93A protein. Hybridoma cells secreting IgG that reacted with apo WT SOD1 but not with native WT SOD1 were selected by ELISA. (B) Confocal microscopic analysis was performed on HeLa cells expressing FLAG-tagged SOD1. Scale bars: 50 μm. (C) Using D3-1 as the first antibody, IHC of the spinal cords from *SOD1^{WT}* mice, *SOD1^{G93A}* mice (arrow denotes mutated SOD1), (D) SOD1 mutated patients with ALS (I112T and C6G), patients with sporadic ALS (sALS) and control (patients with Parkinson's diseases). Scale bars: 500 μm (C) and 25 μm (D). (E) Amino acid map of SOD1 proteins and structural and functional domain information. This epitope may contain a β sheet (β7) and a part of active site loop. (F) Schematic diagram of the three-dimensional structure of SOD1 (PDB: 3T5W). D3-1 epitope (116–123) is colored red and blue in chains A (pink) and B (light blue) of the SOD1 dimer, respectively. The molecular graphics were performed with UCSF Chimera, developed by the Resource for Biocomputing, Visualization, and Informatics at the University of California, San Francisco, with support from National Institutes of Health (NIH) grant P41-GM103311.[19]

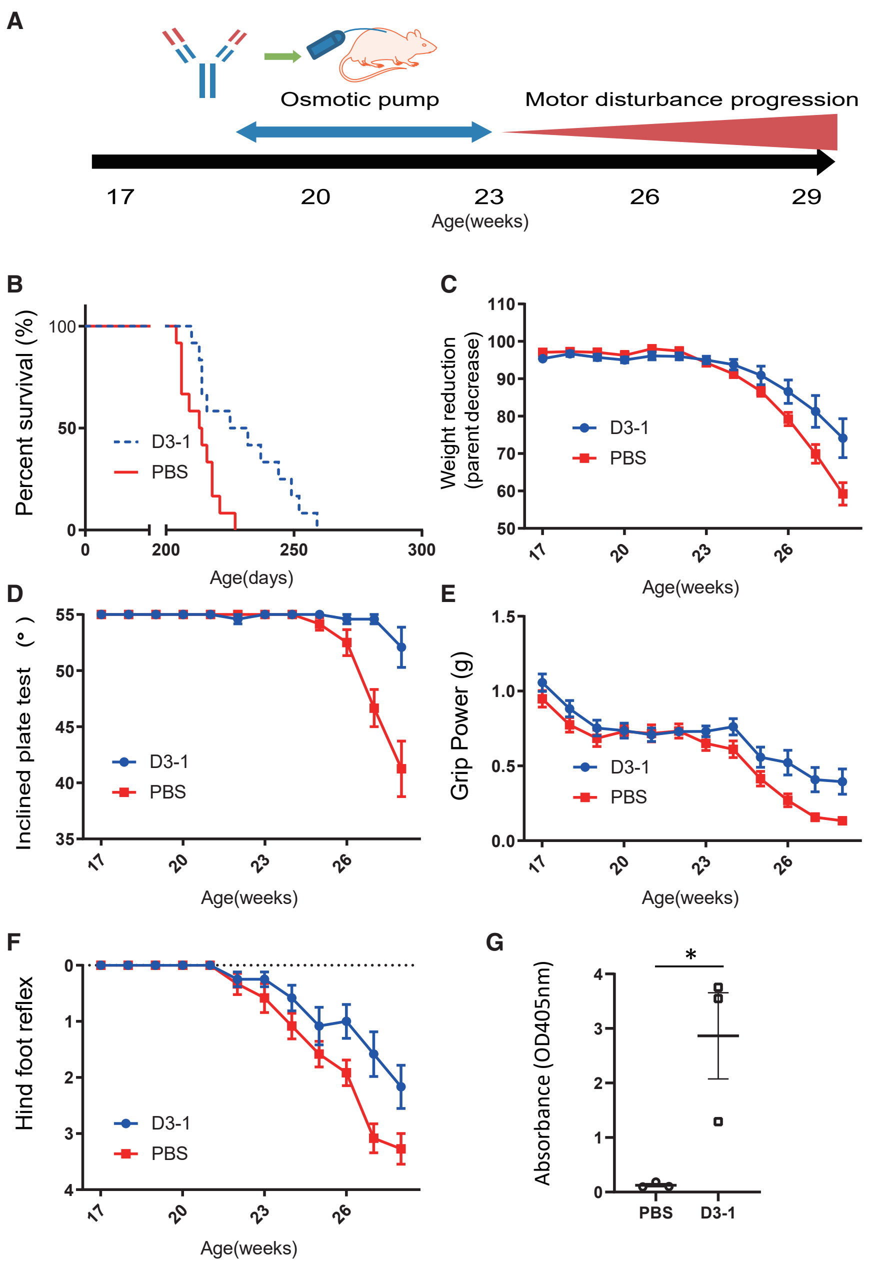

Figure 2. Delayed disease onset and extended lifespan in D3-1 treated-*SOD1*^{H46R} rats

(A) Administration schedule. D3-1 mAb was intrathecally administered to preclinical *SOD1*^{H46R} rats using an osmotic pump for 4 weeks at 19 weeks. (B) Intrathecal administration of D3-1 increased the lifespan of *SOD1*^{H46R} rats. The Kaplan-Meier curve for survival is shown (n = 12, $p < 0.0001$ by log-rank test). (C) Reduction in body weight loss (n = 12, $p < 0.01$). D3-1-treated rats are shown in blue, and PBS-treated rats are shown in red. (D) Inclined plate test, (E) forelimb grip power, and (F) hindfoot reflex score: the score on each test was determined for *SOD1*^{H46R} rats injected with D3-1 and with PBS (n = 12 for all). D3-1 treatment significantly improved motor performance ($p < 0.001$ by post hoc test) compared with PBS-administered rats. Each point indicates average ± SD. Differences were evaluated by two-way ANOVA. (G) The presence of D3-1 mAb in the D3-1-treated rats' CSF after 4 weeks was confirmed by an ELISA. ELISA for a full-length D3-1 in CSF from treated and PBS-injected controls was conducted to confirm the presence of antibodies. Differences were evaluated using Student's *t* test (mean ± SD from three independent experiments; *$p < 0.05$).

rats four weeks after initiating the infusion ($p < 0.05$; mean 2.810 ± 1.192 in D3-1-treated rats versus 0.1264 ± 0.3818 in PBS-treated rats at 4 weeks after administration). To roughly estimate the D3-1 concentrations in the CSF of treated rats, an ELISA was performed using WT rats independently. The concentrations of CSF D3-1 after 4 weeks of osmotic pump administration were 0.485 ± 0.237 μg/mL (Figure S2), which implies comparable concentrations in Tg rats.

Preservation of the antigen specificity of scFv of D3-1 mAb

Subsequently, cDNA for variable fragments of the light chain (VL) and heavy chain (VH) was cloned from D3-1 hybridoma cDNA to generate the scFv of D3-1 (scFvD3-1), as previously reported.[22] The native signal peptides of VH of D3-1 and Myc tag sequences were fused at the amino and carboxyl termini, respectively (Figures 3A and 3B). We performed immunoprecipitation and western blotting (WB) analyses to investigate the mutant SOD1-specific interaction of D3-1 scFv, using the mixture of cell lysates from FLAG-SOD1-transfected cells and the conditioned medium of Myc-D3-1 scFv-transfected cells. We confirmed that scFvD3-1 pulled down only with mutant SOD1 proteins but not with WT, as observed in the full-length D3-1 (Figure 3C). Following confirmation of the antigen specificity of scFvD3-1 and full-length D3-1, we constructed a Borna disease virus vector to transduce scFvD3-1 cDNA into OPCs. BoDV is an RNA virus-based episomal vector that functions as a non-integrating stable gene expression system. We created a defective transmission vector lacking the viral glycoprotein (G) gene (ΔG-REVec). As ΔG-REVec lacks the viral envelope protein, it can initially infect cells and generate viral proteins but does not generate infectious progeny viral particles.[18] GFP was inserted in tandem to visualize the infection efficiency (Figures 3D and 3E). We confirmed that scFv was secreted into the medium of infected OPCs using western blotting. The secretion was endoplasmic reticulum-Golgi mediated, as brefeldin A inhibited extracellular scFvD3-1 (Figure 3F).

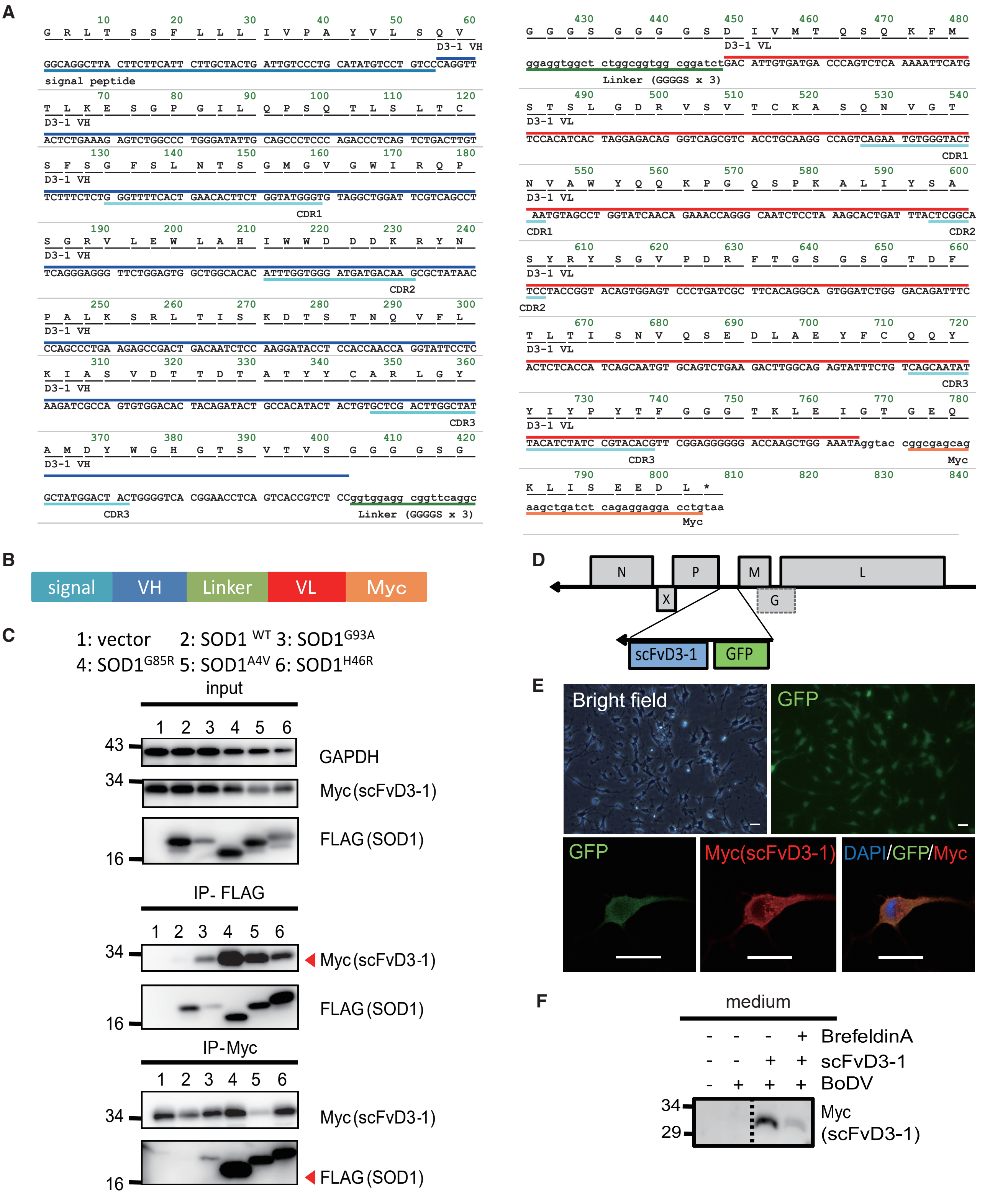

(legend on next page)

Phenotypic improvement of *SOD1*^{H46R} rats by intrathecal injection of scFvD3-1-secreting OPCs

We examined the efficacy of intrathecal transplantation of OPCs with or without scFvD3-1 secretion on the lifespan of rats with ALS. The intracisternal route was chosen to increase cell diffusion in CSF. OPCs expressing secretory scFvD3-1(OPC scFvD3-1) or GFP alone (OPC GFP) were injected intrathecally into *SOD1*^{H46R} rats at the age of 19 weeks (Figure 4A). As a procedural control, the conditioned media of OPC were injected. First, we observed no significant effect of the OPC-GFP on various phenotypic evaluations, including lifespan, body weight, inclined plate test, grip power, and hindlimb reflex, compared with the medium control. In contrast, OPC scFvD3-1 showed a significant extension of average lifespan by 31.5 or 38 days compared with that of OPC-scFv or medium control, respectively (260.5 ± 24.1 versus 229 ± 15.3 versus 222.5 ± 9.8 days in OPC-scFv, OPC alone, and medium, respectively; n = 10, p = 0.0015) (Figure 4B). Moreover, OPC scFvD3-1 suppressed the weight reduction at the age of 26–29 weeks (97.60% ± 13.8% versus 78.16% ± 15.3% versus 75.80% ± 10.6% in OPC-scFvD3-1, OPC alone, and medium, respectively p < 0.0001 at the age of 29 weeks) (Figure 4C). The inclined plate test demonstrated that OPC-scFv-treated animals maintained a significantly longer stay on the higher angle slope compared with those treated with OPC GFP or medium (p < 0.0001; mean 55° ± 0° in OPC scFvD3-1, 43° ± 9.5° in OPC alone, and 43° ± 7.5° in the medium at the age of 29 weeks) (Figure 4D). Grip power revealed a significantly slower decline of muscle strength in rats with OPC scFvD3-1 than those with OPCs alone or with medium control (p < 0.0001; mean 0.6397 ± 0.2341 g in OPC scFvD3-1 versus 0.3054 ± 0.2547 g in OPC alone versus 0.2120 ± 0.0873 g in the medium at the age of 29 weeks) (Figure 4E). Reflex scores, the functional scale of upper motor neurons, demonstrated that OPC scFvD3-1-treated rats showed significantly higher reflex of the hindlimb from 26 to 28 weeks of age compared with those treated with OPC-GFP or control medium (p < 0.001; mean 0.8 ± 1.1 in OPC scFvD3-1 versus 2.6 ± 1.2 in OPC-GFP versus 2.7 ± 0.8 at the age of 29 weeks in the medium) (Figure 4F). Motor function scores of OPC scFvD3-1-treated rats displayed slower decline than those of medium or OPC-GFP-treated rats after 30 weeks of age (Table S2). To estimate whether OPC-scFv therapy surpasses FL D3-1 injection, we compiled data from two different surgical approaches and performed a log rank analysis of the median survival in five groups (12 in each in intrathecal osmotic pumping of FL D3-1 with control, 10 in each in an intracisternal injection of OPC-scFv D3-1 and controls). The results showed that OPC scFvD3-1 achieved a significantly longer survival, as long as 40 days compared with 15 days in intrathecal FL D3-1 injection (p < 0.0001) (Figure 4G). A direct comparison is difficult because the duration of antibody administration and the invasiveness of the surgery are different between 4 week antibody administration and a single transplantation of antibody-secreting cells. We also tested the effect of intraparenchymal injection of these cells into lumbar spinal cords, expecting that transplanted OPCs would provide regenerating effects in the oligodendrocyte surroundings with an aberrantly rapid turnover.[8] However, neither the intraparenchymal injection of OPC GFP nor OPC scFvD3-1 was effective on survival and motor functions (n = 10, p > 0.1) (Figures S3B–S3F). Fluorescent immunostaining revealed that transplanted OPCs with BoDV-GFP and BDV-scFv-GFP were detected 2 days after administration, which decreased gradually after 1 and 3 weeks for both (Figure S3A).

scFvD3-1-expressing OPCs in rat CSF suppressed gliosis and preserved spinal motor neurons and neuromuscular junctions by reducing misfolded SOD1

We further performed IHC for quantitative analysis of the rat spinal cord, using antibodies against choline acetylcholine transferase (ChAT), ionized calcium-binding adapter protein 1 (Iba-1), and glial fibrillary acidic protein (GFAP) for spinal motor neurons, microglia, and astrocytes, respectively. As shown in Figure 5A, OPC-scFvD3-1 prevented the loss of motor neurons and suppressed the proliferation of microglia and astrocytes in the lumbar spinal cords of *SOD1*^{H46R} rats at 25 weeks of age (Figures 5A–5D). We also investigated the effect of OPC-scFvD3-1 on the denervation by evaluating intact neuromuscular junctions (NMJs) at 25 weeks. Sections from the tibialis anterior muscles of *SOD1*^{H46R} rats were co-stained with bungarotoxin (BTX) (binding to the nicotinic acetylcholine receptor of NMJ) and anti-synapshin1 antibody (peripheral synaptic vesicle protein), in which intact NMJs were quantified by counting overlaid spots. The results showed that the OPC scFvD3-1 injection significantly prevented the loss of intact NMJs (Figures 5E and 5F). We investigated the presence of transplanted OPCs immunohistochemically at 3 days and 4 weeks after administration around the cisterna magna. On day 3, OPCs were observed around the pia mater in cisterna magna (Figures 5G and 5H), whereas they were far less detectable 4 weeks later (Figures 5I and 5J). Conversely, the use of sandwich ELISA to investigate scFv activity in the CSF showed a gradual rise in the scFv reactivity until 4 weeks after the injection (Figures 6A and S4). Subsequently, we examined whether OPC-derived scFvD3-1 would provide a quantitative impact on misfolded SOD1 proteins. The tissue homogenates from the lumbar spinal cord of rats 8 weeks after administration were separated into detergent-soluble and detergent-insoluble fractions. Western blotting using an anti-misfolded SOD1 antibody (C4F6) demonstrated that SOD1 was decreased in detergent-soluble and detergent-insoluble fractions (Figures 6B–6D). To investigate whether intracisternal

Figure 3. Generation of an RNA virus-based episomal vector (REVec) carrying scFv D3-1

(A) DNA and peptide sequences of single-chain variable fragments of D3-1 antibody (scFvD3-1) of the heavy chain (VH) (blue line) and light chain (VL) (red line). (B) Domain profiles of scFvD3-1. (C) Immunoprecipitation experiment showing scFvD3-1 secreted by HEK293A cells recognize mutated SOD1 in HEK293A cell lysates. (D) Domain profiles of ΔG-REVec-scFvD3-1 GFP. GFP and scFvD3-1 were inserted between the P and M genes, and the G gene was lacking. (E) Confocal microscopic analysis of OPCs transduced ΔG-REVec -scFvD3-1 GFP showed OPCs were the OPCs expressed GFP and scFvD3-1 sufficiently. Scale bar: 25 μm. (F) Western blotting analysis of the conditioned medium in which OPCs incubated transducted ΔG-REVec -scFvD3-1 GFP showed the OPCs secreted scFvD3-1 in the conditioned medium. Brefeldin A inhibited OPCs secreted scFvD3-1. In (C), (E), and (F), all experiments were conducted at least three times to ensure reproducibility.

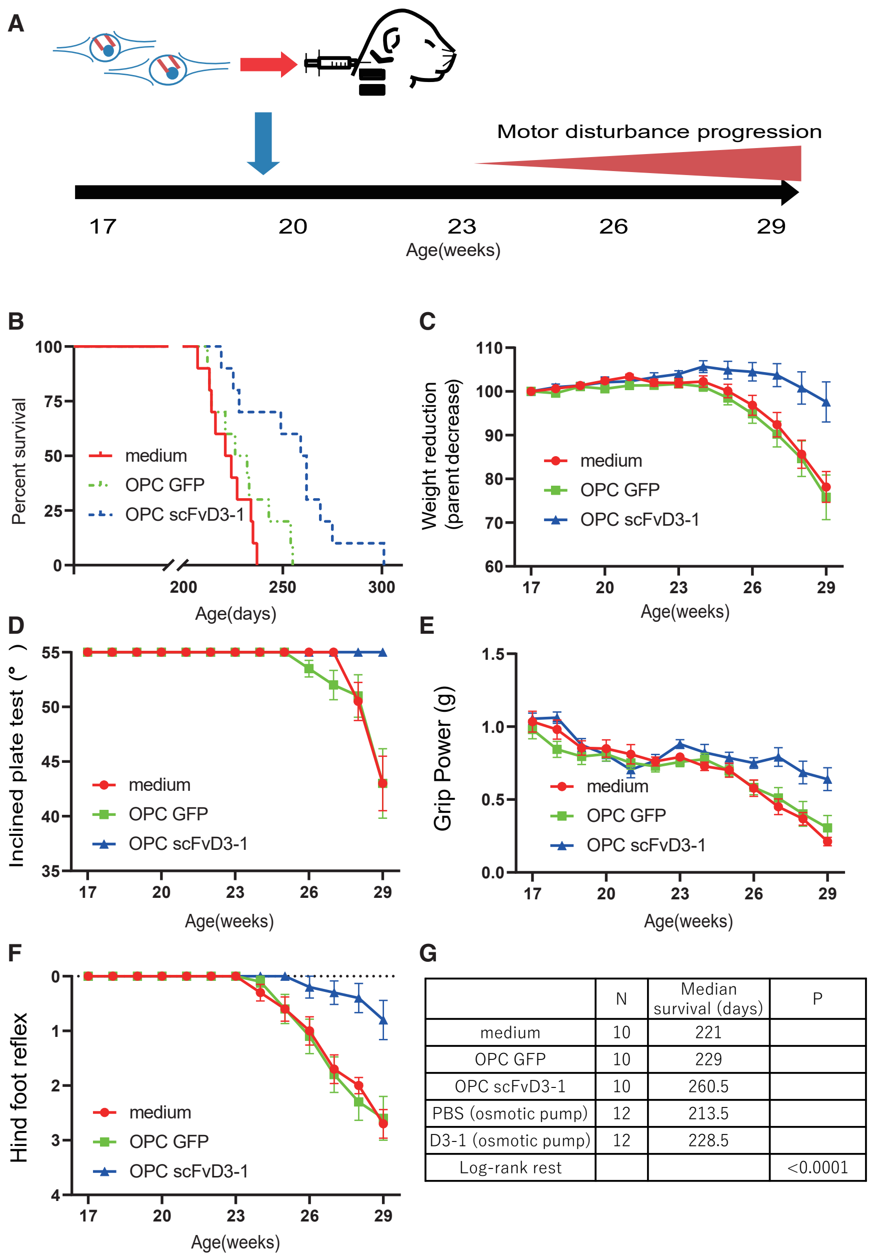

	N	Median survival (days)	P
medium	10	221	
OPC GFP	10	229	
OPC scFvD3-1	10	260.5	
PBS (osmotic pump)	12	213.5	
D3-1 (osmotic pump)	12	228.5	
Log-rank rest			<0.0001

Figure 4. Single intrathecal injection of OPC scFvD3-1 delayed disease onset and increased the lifespan of *SOD1^{H46R}* rats

(A) Transplantation schedule. Intrathecal transplantation was performed on pre-symptomatic *SOD1^{H46R}* rats at 19 weeks of age. (B) Intrathecal administration of OPC scFvD3-1 increased the lifespan of *SOD1^{H46R}* rats. The Kaplan-Meier curve for survival is shown (n = 10 for all, p < 0.0001 by log-rank test) (C) Reduction in body weight loss (n = 10, p < 0.01) OPC scFvD3-1-treated rats are shown in blue, OPC GFP-treated rats are shown in green, and medium-treated rats are shown in red. (D) Inclined plate test, (E) forelimb grip power, and (F) hindfoot reflex score: the score on each test was determined for *SOD1^{H46R}* rats injected with OPC scFvD3-1, OPC GFP, and with medium (n = 10 for all). OPC scFvD3-1 treatment significantly improved motor performances (p < 0.001 by post hoc test) compared with OPC GFP or medium-administered rats. (G) Table showing the mean survival in 5 groups (rats treated with medium, OPC GFP, OPC scFvD3-1 [n = 10], PBS, and D3-1 [osmotic pump, n = 12]). Their survival curves (Figures 2A and 4A) were compared using log-rank test and showed that only the group receiving OPC scFvD3-1 had a statistically superior survival (p < 0.0001). Each point indicates average ±SD. Differences were evaluated by two-way ANOVA.

injection of OPC affects the functions of endogenous oligodendrocytes, we examined the expression levels of MCT-1 in the lumbar spinal cords from treated rats by western blotting since MCT-1 is reportedly decreased in the ALS tissues. MCT-1 was unchanged by the injection of OPC solo or OPC-scFv D3-1 (Figures 6E and 6F).

Microarray analysis revealed that OPC scFvD3-1 improved neuroinflammation via chemokines

To further investigate the molecular mechanisms that endow the therapeutic benefit of the OPC scFvD3-1 transplantation, we performed a microarray analysis of the lumbar spinal cord tissue of *SOD1^{H46R}* rats 8 weeks after the administration of medium, OPC GFP, or OPC scFVD3-1 (n = 3). As shown in the heatmap by function and bubble plot of gene ontology term enrichment, many genes related to inflammation were decreased in rats treated with OPC scFvD3-1 (Figures 7A–7C and S5; Table S3). As oxidized low-density lipoprotein (LDL) receptor 1 (Olr1), chemokine (C-C motif) ligand 2 (*Ccl2*), chemokine (C-X-C motif) ligand 13 (*Cxcl13*), podoplanin (*Pdpn*), Fc fragment of IgG receptor IIb (*FcGR2B*), and TNF alpha-induced protein 6 [(human)] (*Tnfaip6*) showed a remarkable decrease, a real-time qPCR analysis was performed using the same sample. The results showed that *Olr1* and *Tnfaip6* significantly decreased, while other chemokines showed non-significant trends of decrease in the OPC scFvD3-1 compared with GFP OPC or medium control (Figures 7D–7I).

DISCUSSION

In this study, we proposed a new therapeutic paradigm for ALS; the use of OPCs as a delivery vehicle of a therapeutic antibody. Using an amplification-deficient BoDV vector, OPCs were safely transformed to secrete therapeutic scFv targeting misfolded SOD1. BoDV can remain and multiply episomally inside the nucleus of the host cells without affecting genomic integrity, which is an ideal

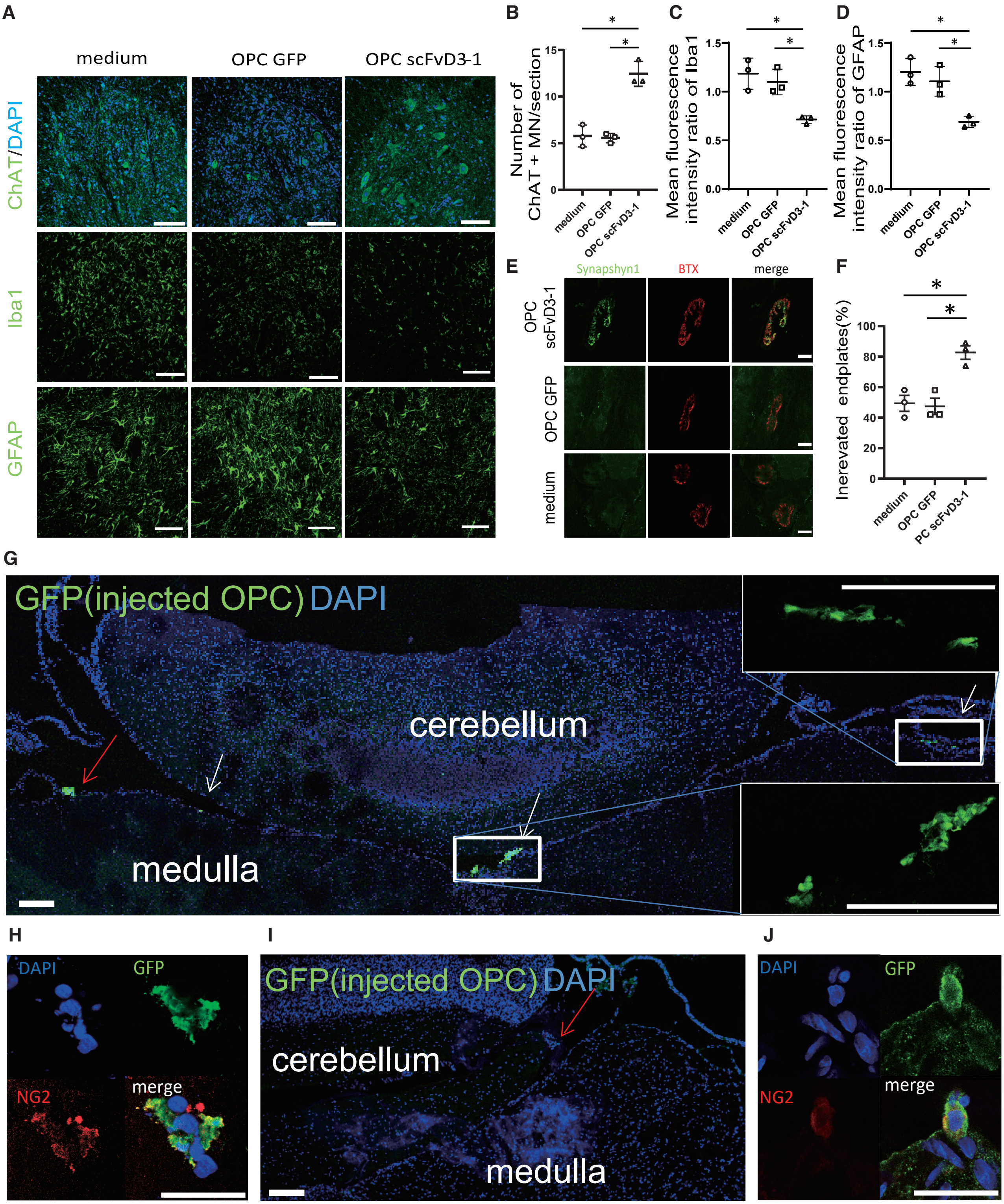

Figure 5. Injection of OPC scFvD3-1 decreased glial activation and neuronal loss of neuromuscular junction (NMJ) denervation in *SOD1^{H46R}* rats

(A) Lumbar spinal cord section from *SOD1^{H46R}* rats treated with medium, OPC GFP, and OPC scFvD3-1 as stained with anti-acetylcholine transferase (ChAT), ionized calcium-binding adapter protein 1 (Iba-1) and glial fibrillary acidic protein (GFAP) antibody. (B) Motor neuron loss was reduced by OPC scFvD3-1 treatment. Quantification of

(legend continued on next page)

viral vector for dividing cells such as OPCs.[16] OPC seems ideal as a carrier of antibody secretion because of its motility and divisibility. Another merit of BoDV is its removability; the antiviral drug favipiravir can eliminate BoVD.[15] Together with a safe and multiplying viral vector such as BoDV, it could apply to other therapeutic strategies. In addition to antibodies,[23] other substances, such as antisense oligonucleotides[22] and growth factors[24] can be secreted, and multiple substances can be secreted as long as they are large enough to fit into a vector. Importantly, direct injection of transmission-free BoDV vector into the rat brain caused no hazardous phenotypes.[25] We did not observe the cytopathic effect of engineered BoDV; however, BoDV negatively affects the production of brain-derived neurotrophic factor (BDNF), an important survival factor for neurons.[26] Moreover, a more precise evaluation is necessary regarding its effect on cell migration or immunogenicity of the recipient cells, which may augment the therapeutic efficacy. The comparison between BoDV and other replicable vectors, such as lentivirus, also deserves consideration.

To date, no report has shown the therapeutic effect of OPC transplantation in ALS model animals, whereas many studies have reported the effects in spinal cord injury models.[27] In our study, the transplantation of OPCs alone in intrathecal space or spinal cord parenchyma failed to ameliorate ALS phenotypes of transgenic *SOD1*^{H46R} rats (Figures 4 and S1). The lack of apparent effect of OPC-solo transplantation may have several explanations, such as poor adhesion or incorporation of OPC cells and the hazardous effects of BoDV. A previous report showed that the oligodendrocyte-specific deletion of mutant SOD1 showed only a limited rescue of the ALS phenotype in mice.[28] Additionally, the neurotoxicity of CSF from patients with ALS has been reported.[29] A previous report documented that CSF toxicity in patients with sporadic ALS was diminished by eliminating misfolded SOD1.[30] MCT-1 dysfunction has been demonstrated in ALS; however,[8] a report showed that the forced expression of MCT-1 in ALS model mice using AAV had no therapeutic effect.[31] The regeneration of OPCs is not sufficient to prevent motor neuron degeneration. Instead, the advantage of OPCs is safety with no concern for tumorigenesis or neuroinflammation.

We attempted two approaches for OPCs transplantation; intrathecal and parenchymal routes. In our study, the administered OPC remained around the cisterna magna for 3 days after the injection. However, OPCs were almost undetectable at 4 weeks after the transplantation, despite the constituted scFv activity (Figure S4). The direct injection of OPCs into the spinal cord parenchyma failed to acquire engraftment. Therefore, the injected OPC may reside in the subarachnoid space, weakly adhering to the pia or arachnoid membranes. This could be why the transplants were hardly detectable using perfusion or rinsing procedures, which implies that the implanted OPCs may have weakly adhered to the subarachnoidal membrane. Another possibility is that the BoDV vector might have affected the adhesion and migration of OPCs, providing an avenue for further investigation. The reason why lower limb functions were preserved despite the lack of remaining OPCs in the lumbar spines is unclear; however, we assume that the diffusion of the scFv throughout the CSF would have provided a therapeutic effect, as reported previously.[5] Considering the augmented benefit of OPC-scFvD3-1 compared with intrathecal injection of a full-length antibody, modification of the transformation to enhance the adhesion of OPCs may further improve the efficacy.

The reason why OPCs were not engrafted in the parenchyma is unclear; however, the extracellular milieu, such as mutant SOD1 might affect the survival of oligodendrocytes. Mutant SOD1 is secreted from various cell types and is implicated in microglial activation or cell-to-cell spreading.[32] Numerous studies have documented the therapeutic benefits of passive or active immunization against misfolded SOD1.[5,10,12,33] Our monoclonal antibody D3-1 specifically recognizes misfolded SOD1. Multiple misfold-specific antibodies have been tried for immunotherapy in ALS models so far[12,13,34–38]; however, not all antibodies have been effective. We adopted several stages of screening processes, including IHC using human and mouse tissues and immunoprecipitation assay. Our results indicate that conformation-conscious selection is effective in obtaining therapeutic antibodies. The misfolded SOD1 in the spinal cord also decreased significantly, which may also contain parenchymal SOD1. A similar result was also reported by Gros-Louis et al.,[12] who showed that secreted SOD1 was incorporated into cells and promoted endosomal proteolysis. This is possible, and we also assume the "sink effect" of extracellular scFv by decreasing extracellular SOD1.

Significantly, oligodendrocytes are actively involved in the immune response.[39] Our transcriptome assay revealed a gross reduction in the expression of inflammation-related genes in the spinal cords of SOD1^{H46R} rats treated with OPC scFvD3-1, in which *Olr1* and *Ccl2* showed a marked decrease. OLR1 and CCL2 proteins reportedly promote neuroinflammation[40,41] by microglial migration and activation.[42,43] Moreover, CCL2 migrates natural killer (NK) cells and accelerates motor neuron (MN) death.[44] Conversely, CCL2 is neuroprotective.[41,42] The exact role of CCL2 in the therapeutic benefit of OPC scFvD3-1 is unclear, and its context-dependent action should be

immunoreactivity score for Iba-1 (C) and GFAP (D) showed a statistically significant reduction of signals in treated rats compared with medium, and OPC GFP (n = 3 for each group; $p < 0.05$, C and D data point was obtained by normalization to the average signal intensity of three groups of rats). (E) Representative immunofluorescent images show neuromuscular junction (NMJ) denervation in SOD1 rats. (F) Quantification of NMJ denervation percentage showed OPC scFVD3-1 administration reduced NMJ denervation compared with OPC, GFP, and medium. (G) Immunofluorescent images of the brainstem and cerebellum around cisterna magna where OPC scFvD3-1 were injected 3 days before. Six clusters of injected OPC were detected (arrows in white and red). Arrow in red indicates the OPC cluster expressing NG2, the higher magnified image of which was shown in (H). (I) Immunofluorescent images of the same region 4 weeks after injecting OPC scFvD3-1. Arrow in red indicates the remaining OPCs, which express NG2, the higher magnified image shown in Figure 5J. All immunostaining was performed on three animals. Approximately 96 sections were prepared per animal, six of which were stained using the antibody of interest. Scale bars: 50 μm (A), 25 μm (E), 100 μm (G), 25 μm (H), 100 μm (I), and 25 μm (J).

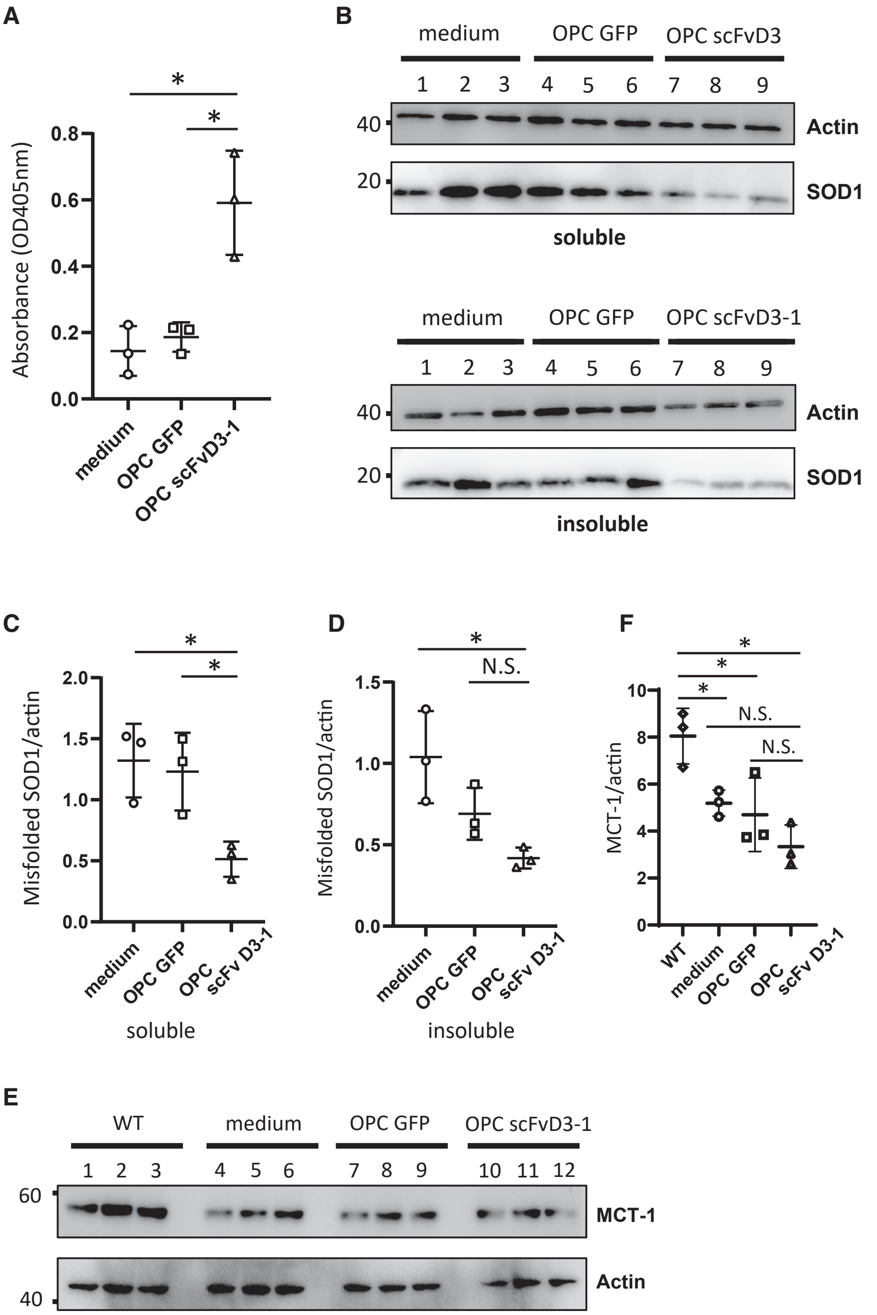

Figure 6. Injection of OPC scFvD3-1 continuously secreted scFvD3-1 and reduced the burden of misfolded SOD1 in *SOD1*^{H46R} rats

(A) The concentration of scFvD3-1 in the cerebrospinal fluid after 4 weeks was measured using sandwich ELISA, using an anti-Myc antibody as a detection antibody. The titer of scFv D3-1 was significantly higher in scFvD3-1-treated rats than in OPC GFP or medium-treated ones. (B) Western blotting analysis showed a reduced misfolded SOD1 in the spinal cord of *SOD1*^{H46R} rats treated with OPC scFvD3-1 (C) Intracysternal injection of OPC scFvD3-1 led to a reduction of ~19% in the levels of misfolded SOD1 species as detected by C4F6 antibody (p = 0.0293). Regarding soluble fractions, the misfolded SOD1 of OPC scFvD3-1-treated rats was significantly lower than that of OPC GFP or medium-treated rats. (D) Regarding insoluble fractions, the misfolded SOD1 of OPC scFvD3-1-treated rats was significantly lower than that of medium-treated ones; however, they were not significantly lower than that of GFP OPC-treated ones. (E and F) Western blotting analysis showed the same amount of MCT-1 in the spinal cord of *SOD1*^{H46R} rats treated with medium, OPC GFP, and OPC scFvD3-1. MCT-1 expression levels were reduced in all treated *SOD1*^{H46R} rats compared with WT rats. Equal amounts of proteins were used, as shown on western blots, after sodium dodecyl sulfate-polyacrylamide gel electrophoresis with an actin antibody. Each data point was obtained by normalization to actin. Data represent mean ± SD. The p value was derived from one-way ANOVA (mean ± SD from three independent experiments; *$p < 0.05$).

further investigated to recognize this two-face molecule as a therapeutic target. OLR1 is the oxidized low-density lipoprotein receptor, activated by oxidized LDL, and is implicated in NF-κB-mediated inflammation.[40] NF-κB reportedly mediates neurodegeneration in TDP-43-linked ALS and is a potential therapeutic target.[45,46] Recent evidence has indicated the relationship between lipid metabolism and pathological conditions in ALS,[47–50] and proposes low LDL as a poor prognostic factor in ALS.[47,48] However, there has been a missing link to provide molecular machinery between the bedside and bench. *Olr1* mutation is a risk factor for Alzheimer's disease, and its downregulation attenuates brain injury in neonatal hypoxic-ischemic encephalopathy in rats.[43,51] Our results indicate that OLR1 contributes to lipid-mediated pathomechanism in ALS.

The main limitation of this experiment was that the survival of transplants was shorter than expected. There is a correlation between the antibody titer and survival time of SOD1 mice.[5] Therefore, repeated injections or improvements in cell culture conditions can augment efficacy in the future. Intravenous administration may be another option for cell transplantation.[7] Besides, there are many unanswered questions regarding the effectiveness of OPC scFVD3-1. Elimination of misfolded SOD1 was observed, and the microarray analyses partly provided several answers regarding the suppression of neuroinflammation. Considering the lack of benefit of OPC solo injection, it is possible that the synergistic effect was attributable to long-term survival to allow perpetual scFv production. Another question is why the parenchymal injection of OPCs-scFv D3-1 was ineffective. As shown

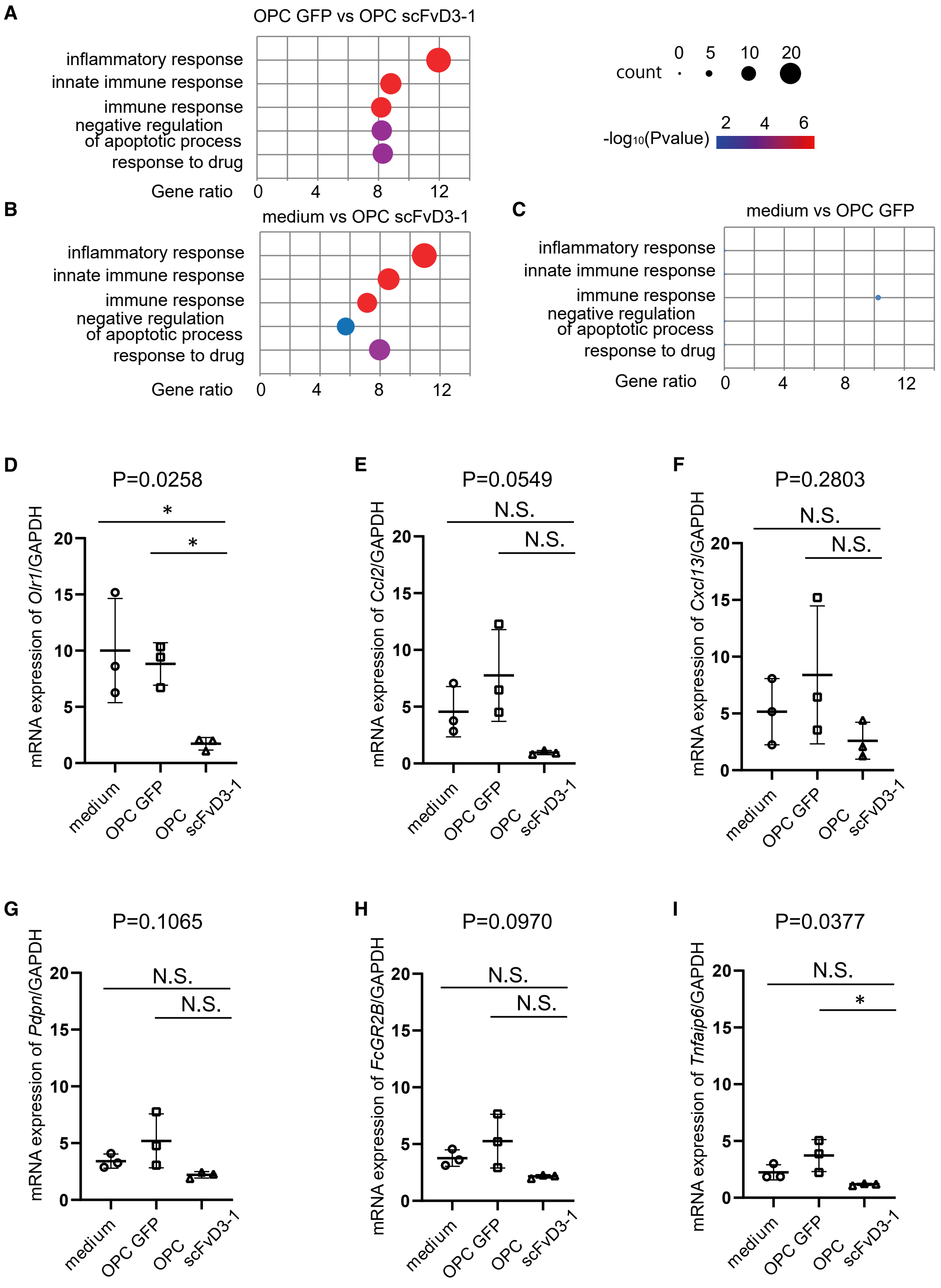

(legend on next page)

in Figure S3, transplants survived for 2 days but almost disappeared 3 weeks after the injection. One possible explanation is that the extracellular milieu affected the survival of the transplants. Another reason might be the immunogenicity of the transformed OPCs. In addition, the miscellaneous effects of the BoDV vector cannot be excluded. It is expected that clarifying these factors may improve the efficacy OPC-scFv therapy.

Furthermore, to apply our results to clinical use, there are several hurdles and challenges. One is the safety of the BoDV vector. There is a technical challenge in obtaining human OPCs from human induced pluripotent stem cells. The safety validation of the OPC transplantation should also be carefully performed. In addition, this study demonstrates the efficacy of treatment in the pre-symptomatic stage of the disease. However, the more critical issue regarding the efficacy of post-symptomatic treatment was not answered, which requires future investigation.

In conclusion, intrathecal transplantation of OPCs secreting therapeutic scFv against misfolded SOD1 is a novel therapeutic paradigm for regenerative immunotherapy. Further investigation of the molecular basis of the efficacy and manipulation to enhance engraftment is required for clinical use.

MATERIALS AND METHODS

Generation and validation of mAb D3-1

The antigen peptide SOD1^{G93A} was injected into C57Bl/6 mice. Hybridomas were generated by fusing mouse splenocytes and P3-X63-Ag8-U mouse myeloma cells using a conventional protocol with 50% polyethylene glycol. The first screening was positive/negative selection against recombinant WT and WT apo SOD1 proteins by ELISA. For epitope mapping, a pGEX-6P-1 vector for expressing glutathione S-transferase (GST)-tagged deleted SOD1 peptide was constructed using the KOD-Plus-Mutagenesis Kit (Toyobo, Osaka, Japan). The GST-tagged deleted SOD1 peptides were expressed in *E. coli* according to the manufacturer's protocol and were purified by eluting with a reduced glutathione solution using an immobilized glutathione Sepharose 4B column (Cytiva). The purified GST-tagged SOD1 peptides were diluted to 1 μg/mL with PBS. One hundred microliters of each peptide were added to each well of 96-well microplates (Maxisorp, Nunc), incubated overnight at 4°C, washed three times with Tris-buffered saline (TBS) containing 0.01% Tween 20 (TBS-T), and then blocked for 2 h at room temperature with 1% BSA in PBS. The plates were washed three times with TBS-T, and 100 μL of mAb D3-1 diluted 1:1,000 or anti-GST (Cytiva) diluted 1:3,000 in TBS-T was added, followed by incubation for 1 h at room temperature. The plates were washed three times with TBS-T, and 100 μL of horseradish peroxidase-conjugated anti-mouse IgG (Promega) or anti-goat IgG (Dako, Kyoto, Japan) diluted 1:5,000 in TBS-T was added and incubated for 1 h at room temperature. After washing five times with TBS-T, the plates were developed using 100 μL of o-phenylenediamine dihydrochloride solution containing 0.015% hydrogen peroxide, and the reaction was stopped with 25 μL of 2 M HCl. The absorbance of each well was determined at 490 nm using a Spectramax spectrophotometer (Molecular Devices). To identify essential amino acid residues for recognition of D3-1, supernatants from *E. coli* extracts expressing GST-tagged deleted SOD1 peptides were applied for immunoblotting using the same antibodies.

Plasmid construction for SOD1

In cell culture studies, mammalian expression plasmids for SOD1 tagged with FLAG (pcDNA3-SOD1-FLAG) were constructed using a conventional PCR technique, as described previously.[52] Several SOD1 substitution mutants with FALS-linked mutations (G93A, G85R, A4V, H46R, and I112M) were generated by site-specific mutagenesis, as previously described. The list of primer pairs is presented in Table S5.

Generation of scFv from D3-1 mAb hybridoma

cDNA was generated from D3-1 hybridoma using mRNA purification kits (Invitrogen, Carlsbad, CA) and cDNA generation (Toyobo) with oligo-dT priming according to the manufacturer's protocol, as previously described.[22] The first-strand cDNAs encoding variable fragment regions of the heavy chain and light chain were amplified separately using the primer pairs for VH and VL (for primer details, see Table S5). The PCR products encoding VH and VL were reacted with TOPO TA cloning vector (Invitrogen) and agarose gel-purified. The VH and VL domains were assembled and linked with a flexible 15-amino acid linker (GGGGS × 3). The double-strand nucleotides were synthesized coding GGGS × 3 linker (5′-GGT GGA GGC GGT TCA GGC GGA GGT GGC TCT GGC GGT GGC GGA TCT-3′ and 5′-AGA TCC GCC ACC GCC AGA GCC ACC TCC GCC TGA ACC GCC TCC ACC-3′). Subsequently, reverse primer spanning VH and the linker (5′-GGC GGT GGC GGA TCT GAG GTT CAG CTG CAG CAG T-3′), and the forward primer spanning the linker and VH, were annealed, which was used as a template for PCR generating VH-linker-VL cDNA, which was subcloned into pCMV-Myc vector (Clonetech) at EcoRI/KpnI sites, and subsequently subcloned into pcDNA3 at EcoRI/XhoI, using the primer pair: 5′-GCC CAG GCC CGA ATT CGC CAT GGA GGT TCA GCT GCA GCA GT-3′ and 5′-AGC TTC TGC TCG CCG GTA CCT ATT TCC AAC TTT GTC CCC-3′.

Cell culture and transfection

All cultured cells were maintained at 37°C under 5% CO_2 and 100% humidity. HEK293A cells (Invitrogen, Carlsbad, CA) were maintained in Dulbecco's modified Eagle's medium (DMEM; Nacalai, Kyoto, Japan) containing 10% fetal bovine serum (FBS) and

Figure 7. Microarray analysis revealed that OPC scFvD3-1 improved immune response via *Orl1*

(A–C) Bubble plot of Gene Ontology (GO) enriched by the differentially expressed genes. About medium versus OPC GFP, 4 of 5 GO term gene counts were 0 (except immune response). (D–I) Real-time qPCR analysis of *Orl1*(D), *Ccl2*(E), *Cxcl13*(F), *Pdpn*(G), *FcGR2B*(H), and *Tnfaip6*(I) gene expression. The differences were evaluated using one-way ANOVA (mean ± SD from three independent experiments; $^{*}p < 0.05$). NS; not significant.

penicillin/streptomycin (Nacalai). Vero cells stably expressing BoDV G (Vero-BG) and Vero REVec-GFP cells were cultured in low-glucose DMEM (Nacalai Tesque, Kyoto, Japan) supplemented with 2% FBS. 293T cells were cultured in high-glucose DMEM (Thermo Fisher Scientific, Waltham, MA) supplemented with 10% FBS. The FuGene HD transfection reagent (Promega) was used for plasmid transfection. At 48 h after transfection, the cells were treated for various analyses, as indicated.

Immunofluorescence and microscopic analysis

Cultured cells were fixed in 4% paraformaldehyde (PFA) in PBS (pH 7.2) and permeabilized with 0.1% Triton X-100/PBS containing 5% normal goat serum as a blocking agent. Cells were incubated with a primary antibody (4°C, overnight) and subsequently with a fluorophore-tagged secondary antibody (Alexa; Invitrogen) for 1 h at room temperature. The cells were counterstained with 4-6 diamidino-2-phenylindole (DAPI). Fluorescence images were obtained using a confocal laser microscope (FV1000-D IX81; Olympus, Tokyo, Japan or SP8, LEICA, Wetzlar, Germany). The antibodies used in this work are described in Table S4.

Western blotting and immunoprecipitation assays

Cultured cells and rat tissues were lysed in immunoprecipitation assay (RIPA) buffer (20 mM HEPES-KOH [pH 7.4], 125 mM NaCl, 2 mM EDTA, 1% Nonidet-P40, 1% sodium deoxycholate) containing a protease inhibitor cocktail (Roche, Basel, Switzerland). The experimental protocols for preparing tissue lysates are indicated later. In several experiments, cultured cells were directly solubilized in sodium dodecyl sulfate (SDS) buffer containing 2-mercaptoethanol for western blotting. In immunoprecipitation, to make cell lysates containing human SOD1, HEK293A cells were transfected with the plasmids for human *SOD1* of WT or several mutants ($SOD1^{A4V}$, $SOD1^{H46R}$, $SOD1^{G85R}$, $SOD1^{G93A)}$. To obtain the conditioned medium of the cells secreting scFv, the same cells were transfected with empty vectors and those for scFvD3-1. Ten percent fractions of SOD1-transfected cell lysates (10 μL) were analyzed as the total cell lysate or input, and the remaining 90% (90 μL) were mixed with 1 mL of the conditioned medium. The lysate-medium mixture was immunoprecipitated with antibody-coated beads against FLAG (Sigma, St. Louis, MI) or Myc (Nacalai) at 4°C. The affinity beads were subsequently washed five times with RIPA buffer, and the immunoprecipitates were eluted in 2% SDS sampling buffer for 5 min at 95°C. The eluates were analyzed using western blotting using anti-Myc or anti-FLAG antibodies to detect the interaction between FLAG-tagged SOD1 and Myc-tagged scFv. The samples were separated on polyacrylamide gels (Wako, Tokyo, Japan), and proteins were transferred onto polyvinylidene difluoride (PVDF) membranes (Millipore, Billerica, MA). Proteins were detected using an enhanced chemiluminescence system (Thermo Fisher Scientific). Densitometric analysis of protein bands was performed using ImageJ software (National Institutes of Health, Bethesda, MD).[53] For western blotting analysis of D3-1 scFv in the conditioned medium transformed OPCs, primary OPCs were infected with BoDV expressing ΔG-REVec-scFvD3-1 GFP and were seeded onto 6-well culture plates (IWAKI) in the OPC nutrient medium as described below. Eight days after infection, brefeldin A (0.2 μg/mL) or dimethyl sulfoxide was added. At 48 h after treatment, 1 mL medium was collected and centrifuged at 1,300 rpm for 3 min at 25°C. After adding 2% SDS sampling buffer, the medium was denatured for 5 min at 95°C. The samples were separated on polyacrylamide gels (Wako), and transferred onto PVDF membranes (Millipore) for western blotting analysis. The enhanced chemiluminescence system was used to detect target proteins (Thermo Fisher Scientific).

Immunohistochemistry of spinal cords from patients with FALS

Postmortem lumbar spinal cords from two patients with neuropathologically-proven *SOD1* mutated ALS (mean age 52.3 years), five patients with sporadic ALS (mean age 67.4 years), and five age-matched control patients (mean age 67.2 years) were analyzed. Control cases included three patients with other neurodegenerative diseases (one with Parkinson's disease; one with dentatorubropallidoluysian atrophy, one with spinocerebellar ataxia), and two without neurodegenerative diseases (one with cerebral infarction; one with epilepsy) (Table S1). During the autopsy, spinal cords were removed, and blocks of the lumbar levels of the spinal cords were immediately placed in 10% buffered formalin and embedded in paraffin. No pathological changes were noted in the spinal cords from control patients. For immunohistochemistry, after heat retrieval by autoclaving (10 min at 121°C in 10 mM sodium citrate buffer), 6-μm-thick sections were incubated overnight at 4°C with D3-1 diluted 1:1,000 with PBS containing 3% BSA (PBS-BSA). Bound primary antibodies were detected with a Vectastain Elite ABC kit (Vector Laboratories, Burlingame, CA) with 3,3′-diaminobenzidine tetrahydrochloride used as the chromogen. The staining specificity was confirmed by replacing the primary antibody with the appropriate amount of PBS-BSA. No reaction product deposits were seen in these control-stained sections. Written informed consent was obtained from all individuals or their guardians before the analysis. The protocols for human sample experiments (R1038) were approved by and performed under the guidelines of the Kyoto University Graduate School of Medicine Ethics Committee. Informed consent was obtained from all individuals or their guardians before the analysis.

Animal breeding and tissue sampling

All Sprague-Dawley rats were obtained from SLC Japan (Shizuoka, Japan). Transgenic rats carrying the human mutant *SOD1* gene for H46*R* ($SOD1^{H46R}$) were housed and genotyped as previously.[21] The DNA of newborn rats was extracted from their tails, and PCR amplification (forward primer: 5′-TTGGGAGGAGGTAGTGATTA; reverse primer: 5′-AGCTAGCAGGATAACAGATGA; 94°C for 30 s, 55°C for 30 s, 72°C for 30 s, 30 cycles) was performed to identify the exogenous human *SOD1* transgene DNA. Founder rats were mated with Sprague-Dawley rats. For western blotting or cDNA microarray experiments, rats were anesthetized intraperitoneally using the combination of medetomidine, midazolam, and butorphanol tartrate. Subsequently, they were intracardially perfused with PBS. For immunohistochemistry, 4% PFA (FUJIFILM Wako Pure Chemical Corporation, Osaka, Japan) was additionally used for tissue fixation.

Preparation of osmotic pumps and transplant surgery

Osmotic pumps (model number 2ML4; Durect Corporation, Cupertino, CA) were incubated in sterile conditions at 37°C for 48 h to attain a constant flow rate before use. Pumps were filled with D3-1 in PBS (1 mg/mL) or PBS using a filling needle. An infusion tube was made by connecting a 1-cm-long polyethylene tubing (PE 60; Becton Dickinson, Franklin Lakes, NJ) to a small caliber tube 9 cm in length (PE 10; Becton Dickinson) using an adhesive (ARON ALPHA; Konishi Co., Osaka, Japan). The end of the infusion tube was connected to the shorter end of the flow moderator, the longer end of which was inserted into the pump. Surgeries for placement of the pump and intrathecal administration were performed as previously described.[26] *SOD1*^{H46R} rats were anesthetized with 2% isoflurane. The skin over the third to the fifth lumbar spinal process was incised, and the paravertebral muscles were separated from the vertebral lamina using scissors. The fifth lumbar vertebra was opened by a laminectomy, and the dura mater was exposed to insert the infusion tube. Special care was taken not to injure the dura mater during the laminectomy. A small hole was bored through the dura mater with a 24G needle, and a polyethylene tube (PE 10; Becton Dickinson) was inserted into the subarachnoid space of approximately 3 cm. A subcutaneous pocket was made into which the osmotic pump and pump side tubes were implanted. The infusion tube was attached to the fascia over the paravertebral muscles at the incision margin with a silk string. A drop of adhesive (ARON ALPHA) was applied, and the incision was closed by suturing the muscles and skin.

Primary oligodendrocyte precursor cell cultures

Mixed glial cell cultures were obtained from the cerebral cortices of 1- to 2-day-old Sprague-Dawley rats and prepared as previously described.[54] Cerebral cortices from 1- to 2-day-old Sprague-Dawley rats were dissected, minced, and digested. Dissociated cells were plated in poly D-lysine-coated 75 cm^2 flasks and maintained in DMEM containing 20% heat-inactivated FBS and 1% penicillin/streptomycin. After the cells were confluent (~10 days), the flasks were shaken for 1 h on an orbital shaker (220 rpm) at 37°C to remove microglia. They were then changed to a new medium and shaken overnight (~20 h). The medium was collected and plated on non-coated tissue culture dishes for 1 h at 37°C to eliminate contaminating astrocytes and microglia. Non-adherent cells were collected and re-plated in a Neurobasal medium containing 2 mM glutamine, 1% penicillin/streptomycin, 10 ng/mL platelet-derived growth factor (PDGF), 10 ng/mL fibroblast growth factor (FGF), and 2% B27 supplement (OPC medium). We have previously reported that the purity of those cells was approximately 99%.[55]

Generation of recombinant REVec and infection into OPCs

ΔG-REVec-scFvD3-1 GFP and ΔG-REVec-GFP were generated as previously described.[18] Transmission-competent REVec was generated by co-transfection of 293T cells with pCAG-BoDV P/M-scFvD3-1 GFP or pCAG-BoDV P/M-GFP vector genome plasmid and helper plasmids (N, P, L, G) using Lipofectamine 2000 (Thermo Fisher Scientific), and overlaid with puromycin-resistant Vero-B cells. At 5 days post-co-culture, cells were treated with puromycin and passaged until most cells became positive for vector production. BoDV-infected Vero-BGcells were suspended in DMEM supplemented with 2% FBS and subjected to sonication. After centrifuging the sonicated cells at 1,200 × g for 25 min at 4°C, the supernatant was collected. Subsequently, 1 mL of 35% sucrose (Nacalai Tesque) in double-distilled water was pipetted into ultra-clear tubes (Beckman Coulter, Brea, CA) and overlaid with the supernatant (1 mL of supernatant, mixed with 2 mL of PBS). Samples were ultracentrifuged at 141,000 × *g* for 1 h at 4°C (Beckman Coulter), and the supernatant was aspirated from the tubes and walls of the tubes dried on paper. Finally, the pellet containing isolated REVec was resuspended in PBS and stored at −80°C. For the gene transduction, OPCs were inoculated with purified REVec three times at 1, 3, and 5 days after plating. After absorption for 1 h, the cells were washed with PBS and maintained in OPC medium. Approximately 70% of the OPCs were available for infection to OPCs after 7 days of plating. The protocols for both genetic transformation experiments were approved by the Kyoto University Graduate School of Medicine Ethics Committee (#163002 for the genetic transformation experiments).

Intrathecal and parenchymal injection of OPCs in SOD1^{H46R}rats

Intrathecal injection of OPCs was performed at postnatal week 19 using *SOD1*^{H46R} rats. A total of 1.0 × 10^6 OPCs-scFv-GFP or OPC-GFP in a 10 μL medium or 10 μL culture medium was injected as a control into cisterna magna using a Hamilton syringe (29G). The syringe was removed after 1 min to minimize CSF and vector leakage. We also investigated the effect of parenchymal injection of OPCs-scFv-GFP, OPC-GFP D3-1, or medium control into bilateral lumbar spinal cords of 19 weeks aged *SOD1*^{H46R} rats. Each rat received two grafts (bilaterally at L3-L4) of 4 × 10^4 cells (in 0.5 μL medium) into the ventral horn. For all surgeries, the rats were anesthetized with 2% isoflurane. After surgery, the animals were housed in cages with free access to food and water until the endpoint. The protocols for the genetic transformation (#29-2 and #29-59) and animal experiments (#2017-5-12 [H3] and #2021-5-15) were approved by and performed under the guidelines of the Shiga University of Medical Science.

Animal assessments of motor functions

The endpoint was defined as the day on which the righting reflex disappeared. Transgenic rats used in the experiments were numbered in birth order. Rats treated with full-length antibodies, D3-1, and PBS were selected numerically and alternately. Rats treated with OPCs were selected numerically in the following order: OPC scFv, OPC GFP, and medium. All functional measurements were performed by a person blinded to the treatment. The methods used were as follows.

Hindfoot reflex test

The animals were lifted by the tail and suspended downward. Extension of the limbs was scored as follows: 0, all limbs fully extended (normal); 1, one limb bent; 2, two limbs bent; 3, three limbs bent; 4, all limbs bent. This test was performed weekly from 17 weeks of age.

Inclined plate test

The animal was placed on the non-slippery wooden slope set at 55°, 50°, 45°, 40°, 35°, or 30° gradient, and the angle at which the animal could remain without slipping for 5 s or more was determined. The test was initiated at 55°. From the second test onwards, measurement started from a higher angle than the maximum angle the animal had tolerated. This test was performed weekly from 17 weeks of age.

Grip test

Forelimb grip strength was determined using a grip strength meter (MK-380Si; Muromachi Kikai, Tokyo, Japan), as previously described,[56] with minor modifications. The rats used their front paws to grab a horizontal bar mounted on the gauge, and the tail was slowly pulled back. Peak tension was recorded when the rats released their grip on the bar. Measurements were repeated five times, and a maximum of five measurements were recorded. This test was performed weekly for 17 weeks.

Immunohistochemistry of animals

Spinal cords were further immersed in 4% PFA PBS solution for 48 h, cryoprotected in 10%–30% sucrose PBS solution, and embedded in Tissue-Tek OCT compound (Sakura Finetek, Tokyo, Japan). Spinal cord or brain stem sections (10–20 μm) were prepared using a Leica cryostat and mounted on MAS-GP glass slides (Matsunami Glass, Osaka, Japan). Sections were immersed in PBS for 5 min, permeabilized in 0.1% Triton X-100 in PBS (0.1% PBS-T) for 30 min, and blocked with Blocking One Histo (Nacalai Tesque) for 10 min. Tissue sections were incubated with primary antibodies diluted in 1% BSA/0.1% PBS-T overnight, washed with 0.1% PBS-T, and incubated with the following secondary antibodies-goat anti-mouse IgG conjugated with Alexa Fluor 555 goat anti-rabbit IgG conjugated with Alexa Fluor 488; donkey anti-goat IgG conjugated with Alexa Fluor 555 (all from Thermo Fisher Scientific). After 1–2 h of incubation with secondary antibodies, tissues were washed with 0.1% PBS-T and mounted in VECTASHIELD HardSet Antifade Mounting Medium with DAPI (Vector Laboratories). Double-immunostained sections were analyzed by confocal microscopy. ChAT-positive motor neurons (with a maximum diameter >20 μm) of the ventral horn of all three lumbar spinal cord slices were manually counted at every 300 μm from the L5 level to obtain the average. Astroglial and microglial activation was quantified by measuring the fluorescence intensity of the confocal images for the GFAP and Iba-1 in the gray matter of all three lumbar spinal cord slices at every 300 μm from the L5 level to obtain the average. The quantification was performed using ImageJ software.

NMJ analysis

Tibialis anterior (TA) muscles were freshly resected and washed in PBS three times. Muscles were fixed with 4% PFA in PBS for 30 min and embedded in Tissue-Tek OCT compound (Sakura Finetek). Muscle sections (40 μm) were prepared using a Leica cryostat and mounted on MAS-GP glass slides (Matsunami Glass). After blocking with the commercially available kit (Blocking One Histo; Nacalai Tesque) for 10 min, the sample slides were incubated with monoclonal antibody anti-synapsin1 (1:200; Sigma-Aldrich) overnight at 4°C. Thereafter, the samples were incubated with a mixture of goat anti-mouse 488 (1:500; Thermo Fisher Scientific) and a 594-conjugated a-bungarotoxin. Fluorescence images along the z axis were acquired using a Leica SPE confocal microscope. Innervated endplates were determined when BTX and synapsyn1 signals co-localized, while the endplates with BTX staining alone were judged denervated. The NMJs were counted until they reached 100 each. Approximately 4−6 slices were investigated in each animal.

ELISA

ELISA was used to measure relative antibody titer in the CSF of rats treated with full-length D3-1 antibody using an osmotic minipump (Alzet pump) or transplanted with OPCs secreting D3-1 scFv tagged with Myc. To capture FL antibody or D3-1scFv-Myc, the recombinant protein SOD1^{G93A} (1 mg/mL) in coating buffer (Roche) was coated onto ELISA plates (Nunc, Rochester, NY) at a 1:100 dilution. 50 μL of CSF from treated rats was applied onto each well and incubated for 1 h at room temperature. After washing with PBS three times, peroxidase-conjugated gout anti-mouse IgG (Jackson Immunoresearch, West Grove, PA, USA) or rabbit anti-Myc antibody (Cell Signaling) was applied to react with full-length D3-1 mAb or D3-1 scFv-Myc (1:500 dilution for both antibodies), and incubated for 1h at room temperature. To detect D3-1 scFv-Myc, peroxidase-conjugated gout anti-mouse IgG antibody (Jackson Immunoresearch) was applied after washing three times with PBS for 16 h at 4°C. Finally, a reaction buffer containing 2,2′-azino-bis-3-ethylbenzothiazoline-6-sulfonate (Roche) was applied, and the absorbance was measured using a spectrometer at 405 nm with a reference wavelength of 490 nm.

Microarray and analysis and real-time qPCR

Total RNA samples were purified from rat spinal cords using a commercially available kit (Invitrogen) and converted to cDNA using reverse transcriptase (Invitrogen). According to the manufacturer's protocol, the collected cDNA was analyzed for microarray using a ClariomTN D assay (Thermo Fisher Scientific). The results were analyzed using the Transcriptome Analysis Console (TAC) software (Thermo Fisher Scientific).

The mRNA expression levels of *orl1*, *ccl2*, *cxcl13*, *pdpn*, *FcGR2B*, *Tnfaip6*, and glyceraldehyde 3-phosphate dehydrogenase (*GAPDH*) were analyzed using a real-time PCR Detection System (Bio-Rad, Hercules, CA) and SYBR quantitative PCR kit (Toyobo) with the following primer pairs: GAPDH was used as an internal standard, and the relative mRNA expression levels were calculated by the ΔΔCT method according to the manufacturer's protocol using the included software (Bio-Rad). The primers used in this study are described in Table S6.

Statistical analysis

For pairwise comparisons, multiple group means were compared using one-way analysis of variance (ANOVA) with post hoc Tukey's

multiple comparison tests. Factor estimation in the two chronological data groups was evaluated by two-way ANOVA using Prism software (GraphPad Software, La Jolla, CA). Statistical significance was set at $p < 0.05$.

DATA AVAILABILITY

The datasets generated during and/or analyzed during the present study are available from the corresponding author upon reasonable request.

SUPPLEMENTAL INFORMATION

Supplemental information can be found online at https://doi.org/10.1016/j.omtm.2023.01.008.

ACKNOWLEDGMENTS

We thank the Central Research Laboratory (CRL) of the Shiga University of Medical Science. We are also grateful for the kind help with the animal housing of members at the Research Center for Animal Life Science (RCALS), Shiga University of Medical Science. This work was supported by a grant-in-aid from the Serika Fund (S.M.), Japan Society for the Promotion of Science (JSPS) KAKENHI KIBAN B 19H03573 (M.U.), and an Intramural Research Grant for Neurological and Psychiatric Disorders (1–5; M.U.) from the NCNP and JST START grant JPMJST211003JA (K.T.) and JSPS KAKENHI 21K19909 (K.T.). The protocols for human sample experiments (R1038) were approved by and performed under the guidelines of the Kyoto University Graduate School of Medicine Ethics Committee.

AUTHOR CONTRIBUTIONS

S.M. performed the experiments, analyzed the data, and wrote the first draft of the manuscript. N.F. contributed to the epitope mapping analysis of D3-1. Hitoshi.Warita. and M.S. provided $SOD1^{H46R}$ rats and thoroughly discussed rat functional assessments. M.S., A.M., and K.T. conducted REVec construction. T.M. contributed to the primary OPC culture. Y.Y. and T.A. contributed to the human histopathological analysis. A.S. contributed to the qPCR analysis. M.K., Hedeki.Wada., R.H., Y.T., M.A., T.M., and R.T. analyzed the data. S.M. and M.U. contributed to the study concept and edited the manuscript. M.U. contributed to the study concept and design and organized all the experiments. All authors read and approved the final manuscript.

DECLARATION OF INTERESTS

The authors declare no competing interests.

REFERENCES

1. Rosen, D.R., Siddique, T., Patterson, D., Figlewicz, D.A., Sapp, P., Hentati, A., Donaldson, D., Goto, J., O'Regan, J.P., Deng, H.X., et al. (1993). Mutations in Cu/Zn superoxide dismutase gene are associated with familial amyotrophic lateral sclerosis. Nature *362*, 59–62.
2. Pramatarova, A., Figlewicz, D.A., Krizus, A., Han, F.Y., Ceballos-Picot, I., Nicole, A., Dib, M., Meininger, V., Brown, R.H., and Rouleau, G.A. (1995). Identification of new mutations in the Cu/Zn superoxide dismutase gene of patients with familial amyotrophic lateral sclerosis. Am. J. Hum. Genet. *56*, 592–596.
3. Eisen, A., Mezei, M.M., Stewart, H.G., Fabros, M., Gibson, G., and Andersen, P.M. (2008). SOD1 gene mutations in ALS patients from British Columbia, Canada: clinical features, neurophysiology and ethical issues in management. Amyotroph Lateral Scler. *9*, 108–119.
4. Miller, T.M., Pestronk, A., David, W., Rothstein, J., Simpson, E., Appel, S.H., Andres, P.L., Mahoney, K., Allred, P., Alexander, K., et al. (2013). An antisense oligonucleotide against SOD1 delivered intrathecally for patients with SOD1 familial amyotrophic lateral sclerosis: a phase 1, randomised, first-in-man study. Lancet Neurol. *12*, 435–442.
5. Patel, P., Kriz, J., Gravel, M., Soucy, G., Bareil, C., Gravel, C., and Julien, J.P. (2014). Adeno-associated virus-mediated delivery of a recombinant single-chain antibody against misfolded superoxide dismutase for treatment of amyotrophic lateral sclerosis. Mol. Ther. *22*, 498–510.
6. Kondo, T., Funayama, M., Tsukita, K., Hotta, A., Yasuda, A., Nori, S., Kaneko, S., Nakamura, M., Takahashi, R., Okano, H., et al. (2014). Focal transplantation of human iPSC-derived glial-rich neural progenitors improves lifespan of ALS mice. Stem Cell Rep. *3*, 242–249.
7. Yamashita, T., Kushida, Y., Wakao, S., Tadokoro, K., Nomura, E., Omote, Y., Takemoto, M., Hishikawa, N., Ohta, Y., Dezawa, M., and Abe, K. (2020). Therapeutic benefit of Muse cells in a mouse model of amyotrophic lateral sclerosis. Sci. Rep. *10*, 17102.
8. Lee, Y., Morrison, B.M., Li, Y., Lengacher, S., Farah, M.H., Hoffman, P.N., Liu, Y., Tsingalia, A., Jin, L., Zhang, P.W., et al. (2012). Oligodendroglia metabolically support axons and contribute to neurodegeneration. Nature *487*, 443–448.
9. Kang, S.H., Li, Y., Fukaya, M., Lorenzini, I., Cleveland, D.W., Ostrow, L.W., Rothstein, J.D., and Bergles, D.E. (2013). Degeneration and impaired regeneration of gray matter oligodendrocytes in amyotrophic lateral sclerosis. Nat. Neurosci. *16*, 571–579.
10. Urushitani, M., Ezzi, S.A., and Julien, J.P. (2007). Therapeutic effects of immunization with mutant superoxide dismutase in mice models of amyotrophic lateral sclerosis. Proc. Natl. Acad. Sci. USA *104*, 2495–2500.
11. Gros-Louis, F., Soucy, G., Larivière, R., and Julien, J.P. (2010). Intracerebroventricular infusion of monoclonal antibody or its derived Fab fragment against misfolded forms of SOD1 mutant delays mortality in a mouse model of ALS. J. Neurochem. *113*, 1188–1199.
12. Poulin-Brière, A., Rezaei, E., and Pozzi, S. (2021). Antibody-based therapeutic interventions for amyotrophic lateral sclerosis: a systematic literature review. Front. Neurosci. *15*, 790114.
13. Briese, T., Schneemann, A., Lewis, A.J., Park, Y.S., Kim, S., Ludwig, H., and Lipkin, W.I. (1994). Genomic organization of Borna disease virus. Proc. Natl. Acad. Sci. USA *91*, 4362–4366.
14. Matsumoto, Y., Hayashi, Y., Omori, H., Honda, T., Daito, T., Horie, M., Ikuta, K., Fujino, K., Nakamura, S., Schneider, U., et al. (2012). Bornavirus closely associates and segregates with host chromosomes to ensure persistent intranuclear infection. Cell Host Microbe *11*, 492–503.
15. Komatsu, Y., Takeuchi, D., Tokunaga, T., Sakurai, H., Makino, A., Honda, T., Ikeda, Y., and Tomonaga, K. (2019). RNA virus-based episomal vector with a fail-safe switch facilitating efficient genetic modification and differentiation of iPSCs. Mol. Ther. Methods Clin. Dev. *14*, 47–55.
16. Daito, T., Fujino, K., Honda, T., Matsumoto, Y., Watanabe, Y., and Tomonaga, K. (2011). A novel borna disease virus vector system that stably expresses foreign proteins from an intercistronic noncoding region. J. Virol. *85*, 12170–12178.
17. Shodai, A., Ido, A., Fujiwara, N., Ayaki, T., Morimura, T., Oono, M., Uchida, T., Takahashi, R., Ito, H., and Urushitani, M. (2012). Conserved acidic amino acid residues in a second RNA recognition motif regulate assembly and function of TDP-43. PLoS One *7*, e52776.
18. Takeuchi, S., Fujiwara, N., Ido, A., Oono, M., Takeuchi, Y., Tateno, M., Suzuki, K., Takahashi, R., Tooyama, I., Taniguchi, N., et al. (2010). Induction of protective immunity by vaccination with wild-type apo superoxide dismutase 1 in mutant SOD1 transgenic mice. J. Neuropathol. Exp. Neurol. *69*, 1044–1056.

19. Pettersen, E.F., Goddard, T.D., Huang, C.C., Couch, G.S., Greenblatt, D.M., Meng, E.C., and Ferrin, T.E. (2004). UCSF Chimera–a visualization system for exploratory research and analysis. J. Comput. Chem. *25*, 1605–1612.

20. Nagai, M., Aoki, M., Miyoshi, I., Kato, M., Pasinelli, P., Kasai, N., Brown, R.H., Jr., and Itoyama, Y. (2001). Rats expressing human cytosolic copper-zinc superoxide dismutase transgenes with amyotrophic lateral sclerosis: associated mutations develop motor neuron disease. J. Neurosci. *21*, 9246–9254.

21. Matsumoto, A., Okada, Y., Nakamichi, M., Nakamura, M., Toyama, Y., Sobue, G., Nagai, M., Aoki, M., Itoyama, Y., and Okano, H. (2006). Disease progression of human SOD1 (G93A) transgenic ALS model rats. J. Neurosci. Res. *83*, 119–133.

22. Amado, D.A., and Davidson, B.L. (2021). Gene therapy for ALS: a review. Mol. Ther. *29*, 3345–3358.

23. Tamaki, Y., Shodai, A., Morimura, T., Hikiami, R., Minamiyama, S., Ayaki, T., Tooyama, I., Furukawa, Y., Takahashi, R., and Urushitani, M. (2018). Elimination of TDP-43 inclusions linked to amyotrophic lateral sclerosis by a misfolding-specific intrabody with dual proteolytic signals. Sci. Rep. *8*, 6030.

24. Ishigaki, A., Aoki, M., Nagai, M., Warita, H., Kato, S., Kato, M., Nakamura, T., Funakoshi, H., and Itoyama, Y. (2007). Intrathecal delivery of hepatocyte growth factor from amyotrophic lateral sclerosis onset suppresses disease progression in rat amyotrophic lateral sclerosis model. J. Neuropathol. Exp. Neurol. *66*, 1037–1044.

25. Komatsu, Y., Tanaka, C., Komorizono, R., and Tomonaga, K. (2020). In vivo biodistribution analysis of transmission competent and defective RNA virus-based episomal vector. Sci. Rep. *10*, 5890.

26. Hans, A., Bajramovic, J.J., Syan, S., Perret, E., Dunia, I., Brahic, M., and Gonzalez-Dunia, D. (2004). Persistent, noncytolytic infection of neurons by Borna disease virus interferes with ERK 1/2 signaling and abrogates BDNF-induced synaptogenesis. Faseb. J. *18*, 863–865.

27. Fu, H., Hu, D., Zhang, L., Shen, X., and Tang, P. (2018). Efficacy of oligodendrocyte progenitor cell transplantation in rat models with traumatic thoracic spinal cord injury: a systematic review and meta-analysis. J. Neurotrauma *35*, 2507–2518.

28. Yamanaka, K., Boillee, S., Roberts, E.A., Garcia, M.L., McAlonis-Downes, M., Mikse, O.R., Cleveland, D.W., and Goldstein, L.S.B. (2008). Mutant SOD1 in cell types other than motor neurons and oligodendrocytes accelerates onset of disease in ALS mice. Proc. Natl. Acad. Sci. USA *105*, 7594–7599.

29. Ng Kee Kwong, K.C., Gregory, J.M., Pal, S., Chandran, S., and Mehta, A.R. (2020). Cerebrospinal fluid cytotoxicity in amyotrophic lateral sclerosis: a systematic review of in vitro studies. Brain Commun. *2*, fcaa121.

30. Tokuda, E., Takei, Y.I., Ohara, S., Fujiwara, N., Hozumi, I., and Furukawa, Y. (2019). Wild-type Cu/Zn-superoxide dismutase is misfolded in cerebrospinal fluid of sporadic amyotrophic lateral sclerosis. Mol. Neurodegener. *14*, 42.

31. Eykens, C., Rossaert, E., Duqué, S., Rué, L., Bento-Abreu, A., Hersmus, N., Lenaerts, A., Kerstens, A., Corthout, N., Munck, S., et al. (2021). AAV9-mediated gene delivery of MCT1 to oligodendrocytes does not provide a therapeutic benefit in a mouse model of ALS. Mol. Ther. Methods Clin. Dev. *20*, 508–519.

32. Hayashi, Y., Homma, K., and Ichijo, H. (2016). SOD1 in neurotoxicity and its controversial roles in SOD1 mutation-negative ALS. Adv. Biol. Regul. *60*, 95–104.

33. Meininger, V., Genge, A., van den Berg, L.H., Robberecht, W., Ludolph, A., Chio, A., Kim, S.H., Leigh, P.N., Kiernan, M.C., Shefner, J.M., et al.; NOG112264 Study Group (2017). Safety and efficacy of ozanezumab in patients with amyotrophic lateral sclerosis: a randomised, double-blind, placebo-controlled, phase 2 trial. Lancet Neurol. *16*, 208–216.

34. Brotherton, T.E., Li, Y., Cooper, D., Gearing, M., Julien, J.P., Rothstein, J.D., Boylan, K., and Glass, J.D. (2012). Localization of a toxic form of superoxide dismutase 1 protein to pathologically affected tissues in familial ALS. Proc. Natl. Acad. Sci. USA *109*, 5505–5510.

35. Maier, M., Welt, T., Wirth, F., Montrasio, F., Preisig, D., McAfoose, J., Vieira, F.G., Kulic, L., Späni, C., Stehle, T., et al. (2018). A human-derived antibody targets misfolded SOD1 and ameliorates motor symptoms in mouse models of amyotrophic lateral sclerosis. Sci. Transl. Med. *10*, eaah3924.

36. Liu, H.N., Tjostheim, S., Dasilva, K., Taylor, D., Zhao, B., Rakhit, R., Brown, M., Chakrabartty, A., McLaurin, J., and Robertson, J. (2012). Targeting of monomer/misfolded SOD1 as a therapeutic strategy for amyotrophic lateral sclerosis. J. Neurosci. *32*, 8791–8799.

37. Dong, Q.X., Zhu, J., Liu, S.Y., Yu, X.L., and Liu, R.T. (2018). An oligomer-specific antibody improved motor function and attenuated neuropathology in the SOD1-G93A transgenic mouse model of ALS. Int. Immunopharmacol. *65*, 413–421.

38. Fujisawa, T., Homma, K., Yamaguchi, N., Kadowaki, H., Tsuburaya, N., Naguro, I., Matsuzawa, A., Takeda, K., Takahashi, Y., Goto, J., et al. (2012). A novel monoclonal antibody reveals a conformational alteration shared by amyotrophic lateral sclerosis-linked SOD1 mutants. Ann. Neurol. *72*, 739–749.

39. Harrington, E.P., Bergles, D.E., and Calabresi, P.A. (2020). Immune cell modulation of oligodendrocyte lineage cells. Neurosci. Lett. *715*, 134601.

40. Pirillo, A., Norata, G.D., and Catapano, A.L. (2013). LOX-1, OxLDL, and atherosclerosis. Mediat. Inflamm. *2013*, 152786.

41. Nardo, G., Iennaco, R., Fusi, N., Heath, P.R., Marino, M., Trolese, M.C., Ferraiuolo, L., Lawrence, N., Shaw, P.J., and Bendotti, C. (2013). Transcriptomic indices of fast and slow disease progression in two mouse models of amyotrophic lateral sclerosis. Brain *136*, 3305–3332.

42. Nardo, G., Trolese, M.C., de Vito, G., Cecchi, R., Riva, N., Dina, G., Heath, P.R., Quattrini, A., Shaw, P.J., Piazza, V., and Bendotti, C. (2016). Immune response in peripheral axons delays disease progression in SOD1G93A mice. J. Neuroinflammation *13*, 261.

43. Akamatsu, T., Sugiyama, T., Oshima, T., Aoki, Y., Mizukami, A., Goishi, K., Shichino, H., Kato, N., Takahashi, N., Goto, Y.I., et al. (2021). Lectin-like oxidized low-density lipoprotein Receptor-1-Related microglial activation in neonatal hypoxic-ischemic encephalopathy: morphologic consideration. Am. J. Pathol. *191*, 1303–1313.

44. Garofalo, S., Cocozza, G., Porzia, A., Inghilleri, M., Raspa, M., Scavizzi, F., Aronica, E., Bernardini, G., Peng, L., Ransohoff, R.M., et al. (2020). Natural killer cells modulate motor neuron-immune cell cross talk in models of amyotrophic lateral sclerosis. Nat. Commun. *11*, 1773.

45. Swarup, V., Phaneuf, D., Dupré, N., Petri, S., Strong, M., Kriz, J., and Julien, J.P. (2011). Deregulation of TDP-43 in amyotrophic lateral sclerosis triggers nuclear factor κB-mediated pathogenic pathways. J. Exp. Med. *208*, 2429–2447.

46. Kumar, S., Phaneuf, D., and Julien, J.P. (2021). Withaferin-A treatment alleviates TAR DNA-binding Protein-43 pathology and improves cognitive function in a mouse model of FTLD. Neurotherapeutics *18*, 286–296.

47. Nakamura, R., Kurihara, M., Ogawa, N., Kitamura, A., Yamakawa, I., Bamba, S., Sanada, M., Sasaki, M., and Urushitani, M. (2021). Prognostic prediction by hypermetabolism varies depending on the nutritional status in early amyotrophic lateral sclerosis. Sci. Rep. *11*, 17943.

48. Nakamura, R., Kurihara, M., Ogawa, N., Kitamura, A., Yamakawa, I., Bamba, S., Sanada, M., Sasaki, M., and Urushitani, M. (2022). Investigation of the prognostic predictive value of serum lipid profiles in amyotrophic lateral sclerosis: roles of sex and hypermetabolism. Sci. Rep. *12*, 1826.

49. Ingre, C., Chen, L., Zhan, Y., Termorshuizen, J., Yin, L., and Fang, F. (2020). Lipids, apolipoproteins, and prognosis of amyotrophic lateral sclerosis. Neurology *94*, e1835–e1844.

50. Guttenplan, K.A., Weigel, M.K., Prakash, P., Wijewardhane, P.R., Hasel, P., Rufen-Blanchette, U., Münch, A.E., Blum, J.A., Fine, J., Neal, M.C., et al. (2021). Neurotoxic reactive astrocytes induce cell death via saturated lipids. Nature *599*, 102–107.

51. Kong, Y., Wu, J.B., Wang, X., Zhao, J.F., Song, H., and Yuan, L.D. (2014). Polymorphism of the OLR1 3′UTR potential microRNA binding site and risk of Alzheimer's disease: a meta-analysis. Genet. Mol. Res. *13*, 10162–10172.

52. Urushitani, M., Kurisu, J., Tsukita, K., and Takahashi, R. (2002). Proteasomal inhibition by misfolded mutant superoxide dismutase 1 induces selective motor neuron death in familial amyotrophic lateral sclerosis. J. Neurochem. *83*, 1030–1042.

53. Schneider, C.A., Rasband, W.S., and Eliceiri, K.W. (2012). NIH Image to ImageJ: 25 years of image analysis. Nat. Methods *9*, 671–675.

54. Maki, T., Takahashi, Y., Miyamoto, N., Liang, A.C., Ihara, M., Lo, E.H., and Arai, K. (2015). Adrenomedullin promotes differentiation of oligodendrocyte precursor cells into myelin-basic-protein expressing oligodendrocytes under pathological conditions in vitro. Stem Cell Res. *15*, 68–74.

55. Yasuda, K., Maki, T., Kinoshita, H., Kaji, S., Toyokawa, M., Nishigori, R., Kinoshita, Y., Ono, Y., Kinoshita, A., and Takahashi, R. (2020). Sex-specific differences in transcriptomic profiles and cellular characteristics of oligodendrocyte precursor cells. Stem Cell Res. *46*, 101866. https://doi.org/10.1016/j.scr.2020.101866.

56. Ono, Y., Maejima, Y., Saito, M., Sakamoto, K., Horita, S., Shimomura, K., Inoue, S., and Kotani, J. (2020). TAK-242, a specific inhibitor of Toll-like receptor 4 signalling, prevents endotoxemia-induced skeletal muscle wasting in mice. Sci. Rep. *10*, 694. https://doi.org/10.1038/s41598-020-57714-3.

OMTM, Volume *28*

Supplemental information

Efficacy of oligodendrocyte precursor cells as delivery vehicles for single-chain variable fragment to misfolded SOD1 in ALS rat model

Sumio Minamiyama, Madoka Sakai, Yuko Yamaguchi, Makiko Kusui, Hideki Wada, Ryota Hikiami, Yoshitaka Tamaki, Megumi Asada-Utsugi, Akemi Shodai, Akiko Makino, Noriko Fujiwara, Takashi Ayaki, Takakuni Maki, Hitoshi Warita, Masashi Aoki, Keizo Tomonaga, Ryosuke Takahashi, and Makoto Urushitani

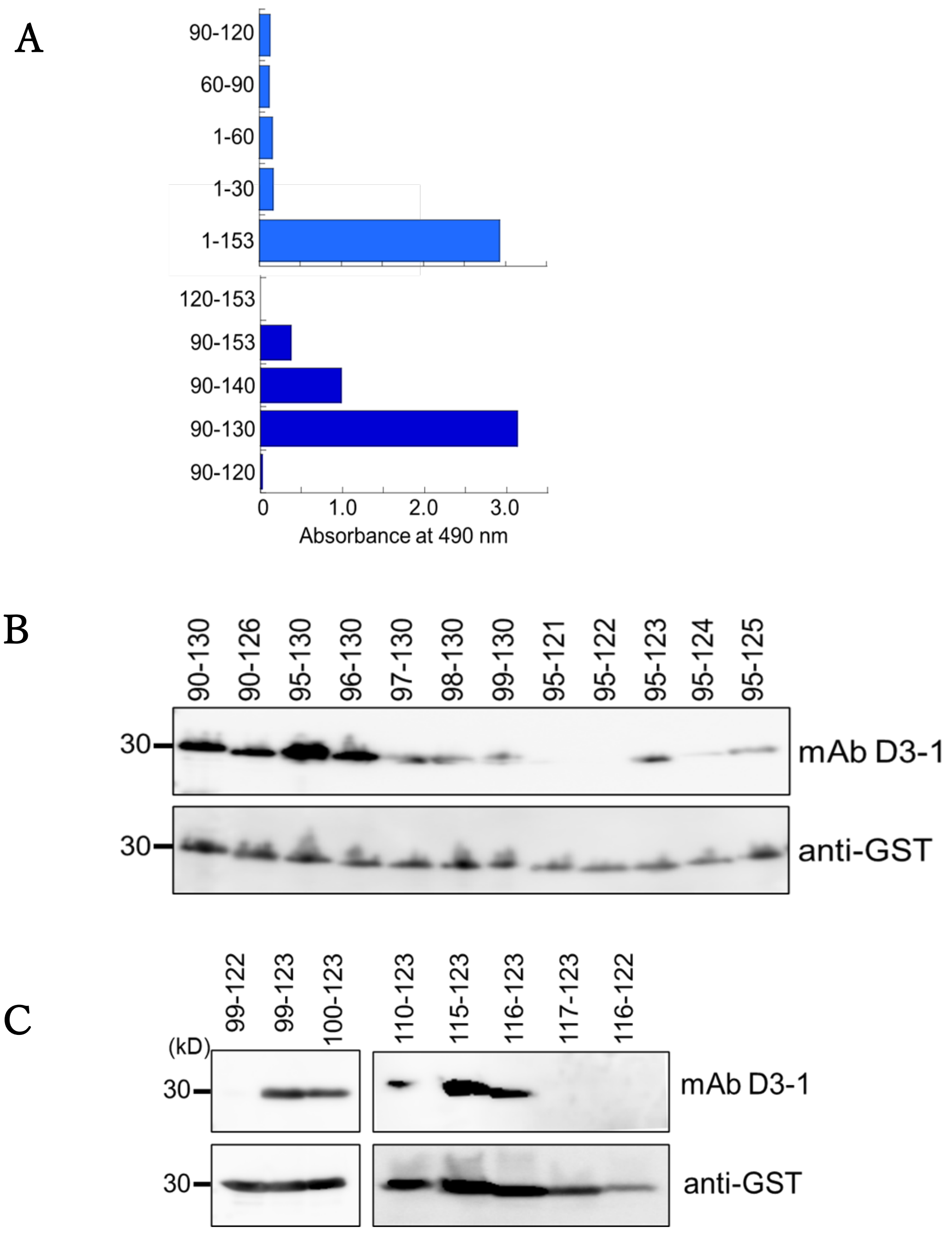

Figure S1. Epitopes of D3-1 exist within residues 116-123 of SOD1. (A) ELISA for GST-tagged deleted SOD1 peptides. **(B) (C)** Immunoblotting of *E. coli* extracts expressing GST-tagged deleted SOD1 peptides.

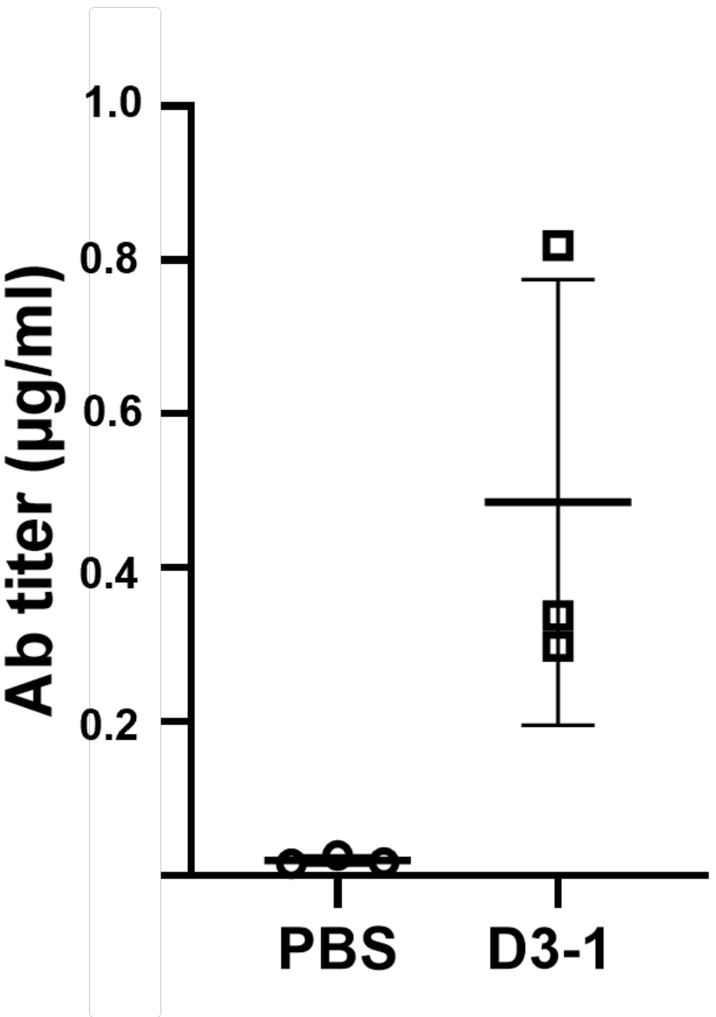

Figure S2. The titer of D3-1Mab in the D3-1 treated wild-type rats' CSF after 4 weeks was confirmed by an enzyme-linked immunosorbent assay (ELISA). ELISA for a full-length D3-1 in CSF from treated and PBS-injected controls was conducted to calculate the titer of antibodies. Differences were evaluated by student t-test (mean ± SD from three independent experiments; * $P < 0.05$).

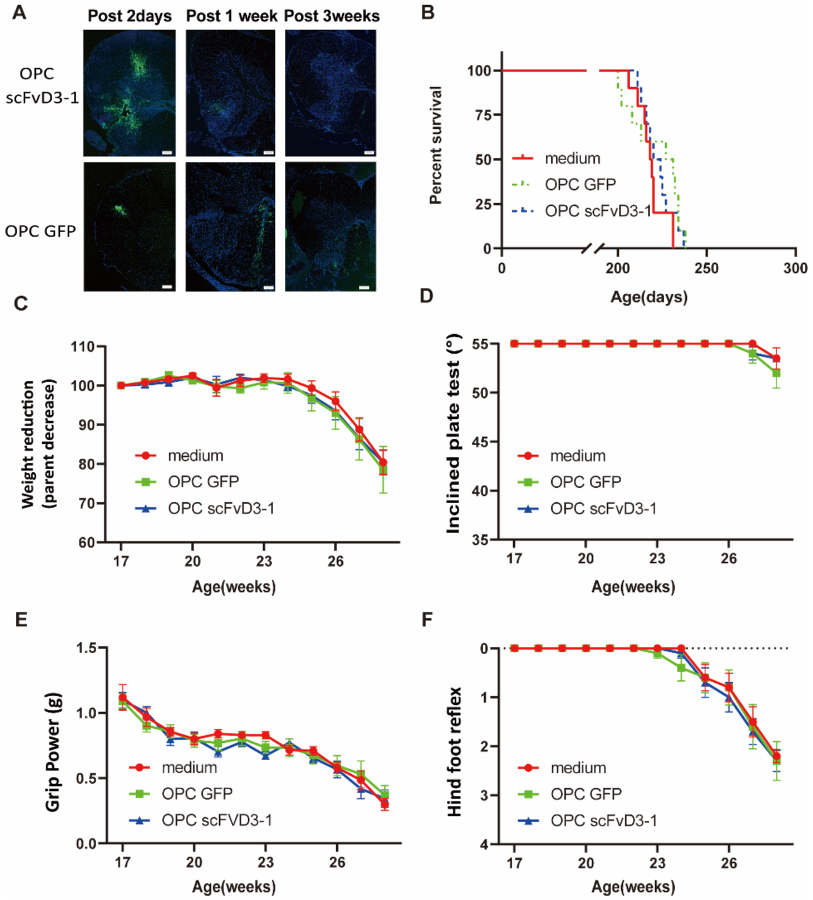

Figure S3. Single parenchymal injection of OPC scFvD3-1 had no therapeutic effect on disease onset and lifespan of SOD1H46R rats. (A) Immunohistochemical images of the lumbar spinal cord of OPC scFvD3-1 or OPC GFP injected rat two days, one week, and three weeks after injection showed a lot of injected alive OPCs two days after injection; however, only a few remained alive one and three weeks after injection. Scale bar 250 µm. (B) Parenchymal administration of OPC scFvD3-1 did not increase the lifespan of SOD1H46R rats. Kaplan–Meier curve for survival is shown (n = 10 for all P > 0.05 by log-rank test). (C) No reduction in body weight loss (P >0.05); OPC scFvD3-1 treated and OPD GFP–treated rats, and medium-treated mice are shown in blue, green, and red respectively. (D) Inclined plate test, (E) forelimb grip power and (F) hindfoot reflex score: the score on each test was determined for SOD1H46R rats injected with OPC scFvD3-1, OPC GFP, and with medium (N =10 for all). OPC scFvD3-1 treatment did not improve motor performance (P > 0.05 by post hoc test) when compared with OPC GFP or medium–administered rats. Each point indicates average ± SEM. Differences were evaluated by two-way ANOVA.

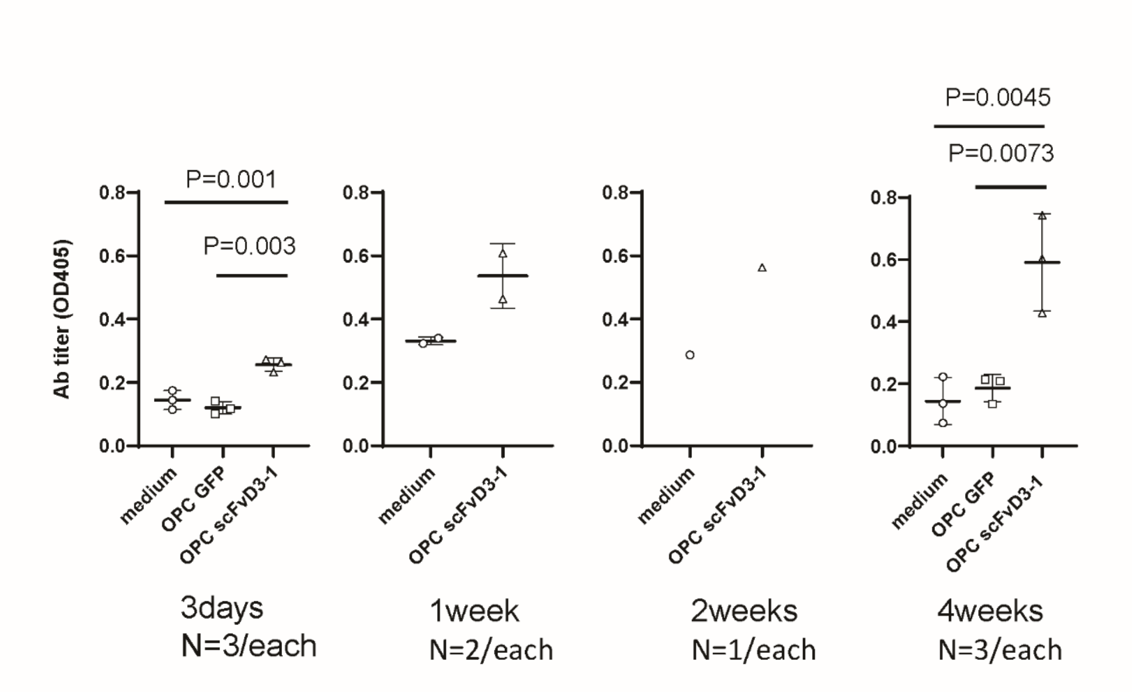

Figure S4. The relative amount of scFv in the cerebrospinal fluid of rats treated with OPC scFv D3-1 increases over time. The concentration of scFvD3-1 in the cerebrospinal fluid at three days one week, two weeks and four weeks after administration of OPC scFvD3-1, OPC GFP or medium, was measured by sandwich ELISA and found to be significantly higher in OPC scFvD3-1 treated rats than in OPC GFP or medium treated ones at three days and four weeks. After 1 and 2 weeks, the values were higher in the OPC scFvD3-1 treated rats than in the medium treated rats.

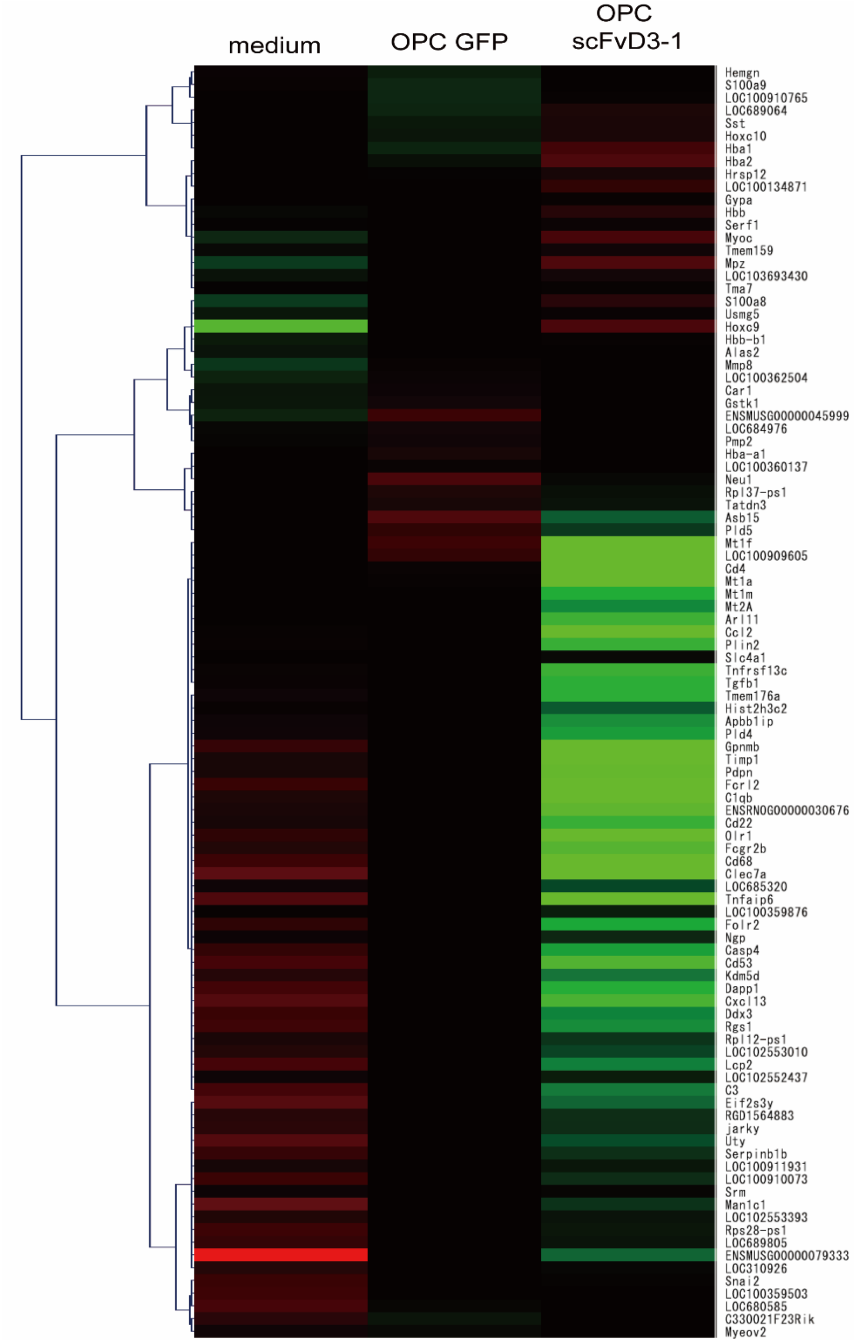

Figure S5. Heat map of microarray analysis using lumber spinal cord of SOD1H46R rats eight weeks after administration of medium, OPC GFP or OPC scFvD3-1(N=3). Green and red lines show decreased and increased genes respectively. The values indicate how far away from the median each of the three groups. The 100 genes with the highest value are listed. Wild-type rats were used as controls.

Table S1 Clinical information of ALS patients and control subjects.

Case no.	sex	Diagnosis	SOD1mutation	Age at death(years)	Duration of the disease
1	Male	mSOD1-ALS	I113T	64	7months
2	Male	mSOD1-ALS	I112T	43	2 years
3	Male	sALS		65	2 years
4	Male	sALS		79	1 years
5	Male	sALS		57	8 years 10 months
6	Male	sALS		65	1 year 11 months
7	Female	sALS		71	1 year 8months
8	Female	C(SCA)		63	
9	Female	C(DRPLA)		59	
10	Male	C(PD)		76	
11	Male	C(CI)		87	
12	Female	C(epilepsy)		51	

nd; not described, m; mutant, s; sporadic, C; control, SCA; spinocerebellar ataxia, DRPLA; dentatorubropallidoluysian atrophy, PD; Parkinson’s disease, CI; cerebral infarction

Table S2 Motor function scores of rats after 29 weeks ages

Table S3 Gene profiles of altered expression levels

Decreased

Gene symbol	Signal value			Z score	
	medium	OPC GFP	OPC scFvD3-1	medium vs. OPCscFvD3-1	OPC GFP vs. OPCscFvD3-1
Gpnmb	6398.686	5409.877	235.688	-22.049	-26.058
Clec7a	2097.115	1232.396	72.245	-24.793	-24.343
Fcrl2	760.392	545.002	40.704	-21.537	-22.255
Cd68	1012.608	740.457	106.018	-16.581	-16.644
Tnfaip6	607.425	440.746	77.010	-15.167	-14.927
Ccl2	568.497	438.094	83.077	-14.117	-14.221
Olr1	513.551	421.769	89.707	-12.799	-13.232
Mt1a	12055.217	12279.805	2283.657	-10.641	-13.138
Mt1f	320.601	390.308	90.946	-9.217	-12.445
Tmem52b	180.636	112.667	51.997	-17.607	-12.413
C1qb	3065.082	2273.895	510.079	-11.951	-12.369
Cd4	20494.086	20494.086	4381.746	-9.864	-12.038
Ccl3	46.152	46.152	22.406	-10.187	-11.595
Mpeg1	4460.254	3880.186	997.905	-9.970	-11.227
Cd22	143.984	134.326	66.928	-10.804	-11.176
Csf2rb	113.498	113.498	56.974	-9.713	-11.055
Pdpn	17133.829	15036.279	3650.909	-9.886	-11.035
Cyp4v3	456.240	475.367	131.806	-9.082	-10.945
Glipr1	171.043	149.902	77.420	-5.765	-10.596
Grn	2582.495	2334.439	662.776	-9.051	-10.401
Cxcl13	2087.065	1229.444	369.978	-11.528	-9.914
Cd53	16982.250	11499.552	3278.295	-10.520	-9.770
Fcgr2b	2680.804	2082.918	643.658	-9.497	-9.693

Increased

Gene symbol	Signal value			Z score	
	medium	OPC GFP	OPC scFvD3-1	medium vs. OPCscFvD3-1	OPC GFP vs. OPCscFvD3-1
Igkc	93.805	78.997	391.847	10.653	13.922
Chodl	2249.268	3873.065	11973.356	10.765	9.026
Myh4	36.889	36.889	60.771	7.156	8.134
Slc18a3	742.445	1392.421	2945.323	9.283	6.376
Ahnak2	13863.462	14418.565	25476.156	3.809	5.349
Susd2	1381.567	1832.643	3399.696	6.086	4.999

Table S4 Antibodies used in the study

	Applications	Host	Source	catalog #
C4F6	WB	Mouse	Medimabs	#MM-0070-2
FLAG	WB,IF,IP	Mouse	Sigma-Aldrich	F1804
β-actin	WB	Mouse	Santa Cruz	sc-47778
Myc	WB,IF,IP,ELISA	Rabbit	Cell Signaling	#2278
GAPDH	WB	Rabbit	Santa Cruz	sc-25778
GFP	IF,IHC	Rabbit	MBL	598
chat	IHC	Goat	Millipore	AB144P
iba1	IHC	Rabbit	Wako	MNH4488
GFAP	IHC	Mouse	Sigma-Aldrich	032M4779
synapshin1	IHC	Mouse	Sigma-Aldrich	S5788
NG2	IF	Mouse	abcam	ab5009
α-Bungarotoxin	IHC		ThermoFisher	B13423
MCT-1	WB	Rabbit	Millipore	AB3540P

WB: western blot; IF: Immunofluorescence; IHC: immunohistochemistry;

IP: immunoprecipitation; ELISA: Enzyme-linked immunosorbent assay

Table S5 Primer Sequences for the construction

SOD1 I112M fwd	5'-TCA GGA GAC CAT TGC ATG ATT GGC CGC ACA C-3'
SOD1 I112M rev	5'-GTG TGC GGC CAA TCA TGC AAT GGT CTC CTG A-3'
SOD1 H46R fwd	5'-GGG GCT AGC CAC CAT GGC GAC GAA GGC CGT GTG-3'
SOD1 H46R rev	5'-CCC GGA TCC CGT TGG GCG ATC CCA ATT ACA C-3'
VH fwd	5'-GAC TCG AGT CGA CAT CGA TTT TTT TTT TTT TTT TT-3
VH rev	5'-CTC AAT TTT CTT GTC CAC CTT GGT GC-3'
VH fwd	5'-GAC TCG AGT CGA CAT CGA TTT TTT TTT TTT TTT TT-3'
VL rev	5'-CTC ATT CCT GTT GAA GCT CTT GAC AAT GGG-3'

Table S6 Primer sequences for quantitative real-time PCR

Gene name	Primer Sequence
Ccl2_F	CTATGCAGGTCTCTGTCACGCTTC
Ccl2_R	CAGCCGACTCATTGGGATCA
Pdpn_F	TGAGGCTCCAACGAGATCAAGA
Pdpn_R	CGCTGAGTCCCAGAACCAAAC
Cxcl13_F	AACTCCACCTCCAGGCAGAATG
Cxcl13_R	GGTCGAGCTCACCTTGGAACA
Fcgr2b_F	CAGCAACAGGACTGTCGTCCA
Fcgr2b_R	GCTCAAGTTTCACCACAGCCTTC
Olr1_F	GATGACAAGATGAAGCCTGTGAATG
Olr1_R	GGATGGCCAGAGTCACAGCA
Tnfaip6_F	CGTCTTGCAACCTACAAGCAGCTA
Tnfaip6_R	ACAGTTGGGCCCAGGTTTCA
GAPDH_F	CAAGTTCAACGGCACAGTCAAG
GAPDH_R	ACATACTCAGCACCAGCATCAC
